# Improved mapping of Arctic fractional land cover and land cover change from multi-resolution optical remote sensing

**DOI:** 10.1016/j.rse.2026.115511

**Published:** 2026-10

**Authors:** Xiaoran Zhu, Jonathan A. Wang, Oliver Sonnentag, Isla H. Myers-Smith, Daryl Yang, Kathleen M. Orndahl, Leon Nill, Antoine Caron-Guay, Etienne Laliberté, Mark A. Friedl

**Affiliations:** a Department of Earth and Environment, Boston University, Boston, MA, USA; b School of Biological Sciences, University of Utah, Salt Lake City, UT, USA; c Département de géographie, Université de Montréal, Montréal, QC, Canada; d Department of Forest & Conservation Sciences, University of British Columbia, Vancouver, BC, Canada; e Environmental Sciences Division, Oak Ridge National Laboratory, Oak Ridge, TN, USA; f School of Informatics, Computing, and Cyber Systems, Northern Arizona University, Flagstaff, AZ, USA; g Geography Department, Humboldt-Universität zu Berlin, Unter den Linden 6, Berlin, Germany; h Institut de recherche en biologie végétale, Université de Montréal, Montréal, QC, Canada

**Keywords:** Arctic vegetation, Remote sensing, Land cover change, Shrubification, Land surface phenology, HLS, High-resolution satellite imagery

## Abstract

Changes in Arctic tundra vegetation, driven by climate change, may be inducing major shifts in ecosystem services and the Arctic carbon budget, and altering high latitude feedbacks to the climate system. Field-based studies have documented warming-induced shrub expansion, and remote sensing has revealed heterogeneous, but primarily positive, trends in peak summer greenness across the Arctic. However, efforts to move beyond remotely sensed measures of spectral greening to quantify the spatial extent and rate of shrub expansion have been constrained by spectral similarities among tundra vegetation types, limited ground truth data, low revisit frequency of satellite observations, and sub-pixel heterogeneity of land cover at medium spatial resolution (30 m). To address these challenges, we developed a methodology that integrates high spatial resolution (2 m) commercial satellite imagery with Harmonized Landsat and Sentinel-2 observations in a machine learning framework, and used it to produce annual maps for 2016 to 2023 of sub-pixel land cover fractions at 30-m spatial resolution across three Arctic tundra ecoregions spanning 3.35 × 10^5^ km^2^ between the Seward and Tuktoyaktuk Peninsulas. Uncertainty was quantified at each pixel via Monte Carlo resampling. Independent accuracy assessments yielded good accuracies (mean squared errors of 15.98% and 11.89% for low-stature vegetation and erect shrub cover, respectively), that were comparable to or exceeded previous mapping efforts. Further, repeat commercial satellite image pairs enabled the first assessment of mapped fractional cover change in Arctic tundra (R^2^ of 0.46 and 0.55, change direction accuracies of 77% and 78% for low-stature vegetation and erect shrub cover, respectively). This novel, scalable, multi-sensor approach to fractional land cover mapping produced the first annual maps of land cover fractions in the Arctic tundra, which support more accurate representation of vegetation dynamics and their linkages to climate change and disturbance processes.

## Highlights

• The first annual sub-pixel (30 m) land cover maps for Arctic tundra were produced.
• Phenology-informed spectral-temporal features improved land cover modeling.
• High-res satellite data enabled scalable training and a first assessment of change.
• Pixel-level uncertainty estimates support interpretation and downstream use.
• Results capture disturbance-driven transitions and gradual vegetation change.

## 1 Introduction

Arctic amplification of climate change, leading to warming at up to four times the global average rate, is rapidly altering environmental conditions in regions characterized by some of the lowest temperatures and shortest growing seasons across all terrestrial biomes (Box et al., 2019; Rantanen et al., 2022). Changing climate patterns have also led to increasing frequency and magnitude of disturbance regimes, most notably through intensifying wildfires and abrupt permafrost thaw events (Descals et al., 2022; Foster et al., 2022; Natali et al., 2021; Webb et al., 2025). Both long-term climate change and pulse disturbances are altering the composition, structure, and function of tundra vegetation (Box et al., 2019), which have the potential to drive cascading impacts on local (e.g., subsistence and cultural activities), regional (e.g., water regulation) and global ecosystem services (e.g., carbon regulation) (Chapin et al., 2005; Mekonnen et al., 2021; Virkkala et al., 2025).

Widespread changes in Arctic tundra vegetation have long been documented from time series of remotely sensed spectral vegetation indices such as the normalized difference vegetation index (NDVI), which is widely used as a proxy for vegetation productivity. From the earliest reports derived from AVHRR (Myneni et al., 1997) to more recent accounts that resolve finer spatial patterns using MODIS (Frost et al., 2025; Frost et al., 2023; Myers-Smith et al., 2020) and Landsat (Berner et al., 2020; Frost et al., 2025; Ju and Masek, 2016; Wang and Friedl, 2019), studies have identified heterogeneous trends dominated by increases in spectral vegetation indices such as the NDVI (hereafter, spectral greening) (Myers-Smith et al., 2020), ostensibly driven by warming and mediated by regional and local variations in climate, topography, hydrology, permafrost, and disturbance regimes (Berner et al., 2020; Frost et al., 2025).

Field-based studies examining changes in tundra vegetation have largely focused on shrubification, the densification and expansion of erect shrubs in response to warming, as a key mechanism that explains spectral greening in the Russian Arctic (Forbes et al., 2010), Western Canadian Arctic (Fraser et al., 2014), and at numerous other sites across the pan-Arctic (Elmendorf et al., 2012; Mekonnen et al., 2021). Shrubification is of critical importance because it alters a wide array of tundra environmental properties including wildlife habitat, snow cover, permafrost dynamics, nutrients cycling, and carbon balance, while increasing fuel load and decreasing surface albedo. These changes also often promote shrubification and trigger positive climate feedbacks (Foster et al., 2022; Grünberg et al., 2020; Mekonnen et al., 2021).

While the expansion of erect shrubs represents one of the key ways that Arctic landscapes are changing, shrubs have expanded primarily in riparian zones, floodplains, hillslopes, and recently-disturbed areas (Frost et al., 2025; Lantz et al., 2009). Spectral greening trends, however, are more widespread and likely reflect a broad range of land surface processes including long-term changes in vegetation cover and composition, increases in leaf area and productivity of existing vegetation, and changes in surface water extent, snowmelt timing, and disturbance (Frost et al., 2025; Myers-Smith et al., 2020). Field- and remote sensing-based studies have found that greening has also occurred at sites dominated by graminoids, other forms of herbaceous vegetation, and in areas of bare ground or sparse vegetation (Elmendorf et al., 2012; Ju and Masek, 2016; McManus et al., 2012; Mekonnen et al., 2021; Wang and Friedl, 2019). Additionally, disturbance events lead to both local browning and greening from vegetation recovery following release of nutrients and active layer deepening (Foster et al., 2022; Myers-Smith et al., 2020), all of which add complexity to the processes that are driving trends in spectral greening. Hence, greenness trends reflect multiple modes of ecological change that are occurring in the Arctic. Because of this, a need exists to better understand the ecological processes being captured by spectral greening and browning, and to better quantify the types, extent, and drivers of Arctic land cover change (Bjorkman et al., 2020; Myers-Smith et al., 2020). Achieving this goal, however, is difficult for three main reasons.

First, because tundra vegetation communities are heterogeneous across short length scales (< 30 m), discrete maps of land cover derived from medium spatial resolution imagery cannot capture local variation in tundra vegetation (Beamish et al., 2020). To address this, recent Arctic-specific mapping efforts have used regression models to estimate the fractional cover of different vegetation types at sub-pixel resolution (Beck et al., 2011; He et al., 2019; Macander et al., 2022; Nill et al., 2022). However, these efforts vary in how they define vegetation classes, ranging from broad groupings such as “woody” versus “herbaceous” to more granular plant functional types (PFTs). While finer distinctions are ecologically meaningful, spectral similarities among low-stature tundra PFTs make cover fractions difficult to resolve within 30-m pixels without hyperspectral imagery (Beamish et al., 2020; Domine et al., 2025; Yang et al., 2023). Definitional variability and limited spectral separability introduce uncertainty and disagreements among existing products (He et al., 2019).

Second, the geographic extent and remoteness of the Arctic present significant challenges for collection of training and reference data used to estimate models and assess land cover maps derived from remote sensing (Beamish et al., 2020). While recent synthesis efforts have sought to harmonize vegetation cover data from independent field studies (Berner et al., 2024; Droghini et al., 2025; Steckler et al., 2025; Zhu et al., 2024), and plot data has been used to map continuous land cover fractions in the Arctic (He et al., 2019; Macander et al., 2022), traditional plot-based sampling methods are not well-suited for satellite-based fractional cover mapping because of scale mismatches between plot data (often 1 m^2^) and remotely sensed medium resolution pixels (900 m^2^). Further, plot sizes, estimation techniques, and geolocation accuracy vary widely between independent studies (Berner et al., 2024; Zhu et al., 2024). Observations collected using unmanned aerial vehicles (Assmann et al., 2019; Yang et al., 2022) and techniques such as ‘pixel-walking’ (Wong et al., 2024) are valuable at local scales, but are labor-intensive and geographically limited, and collecting repeat measurements in support of efforts to assess the quality of maps depicting changes over time is often impractical. As a result, assessing the accuracy of existing regional maps depicting sub-pixel changes in Arctic vegetation and land cover is not currently possible (Macander et al., 2022; Nill et al., 2022).

Third, long-term change analysis at moderate spatial resolution has relied on Landsat imagery (Wulder et al., 2019). However, the density of Landsat imagery across the Arctic is relatively sparse, especially in Alaska prior to 2000 (Zhang et al., 2022), constraining existing studies to mapping land cover by pooling data across multiple years (Beck et al., 2011; He et al., 2019; Macander et al., 2022, Macander et al., 2017; Nill et al., 2022). While such studies provide estimates of gradual, long-term land cover change, maps derived from multi-year data do not capture pulse changes related to disturbances (Macander et al., 2022; Nill et al., 2022) and therefore are unable to capture the timing, magnitude, and frequency of disturbance events, which play a large and growing role in facilitating shrubification (Foster et al., 2022). As disturbance events intensify and become more frequent under climate change, it will be increasingly important to quantify and attribute abrupt changes in land cover, which can catalyze transitions in vegetation regime (Chen et al., 2021a; Gaglioti et al., 2021).

To address these challenges, we present a novel methodology that integrates high spatial resolution reference data collected from commercial satellite imagery with high temporal resolution information from Harmonized Landsat and Sentinel-2 imagery (HLS; Claverie et al., 2018) to map sub-pixel land cover fractions at annual timestep in Arctic tundra ecosystems. Our framework combines the unique strengths of these sensors to map the fractional cover of five key land cover classes at medium (30 m) spatial resolution: water bodies, barren cover, low-stature vegetation (henceforth, low vegetation), erect shrubs (henceforth, shrubs), and trees. In developing these methods and analyzing change, our analysis examines the following questions:

1) How effectively can continuous fractional composition of land cover be mapped using a combination of commercial high-resolution imagery and spectral-temporal features derived from HLS?
2) How do model uncertainty estimates relate to spatial patterns of land cover and prediction error?
3) How accurately do mapped changes capture observed land cover change from known disturbances and repeat commercial high-resolution imagery observations?

## 2 Study area

The study area encompassed 3.35 × 10^5^ km^2^ and includes three level III ecoregions classified as tundra in the Commission for Environmental Cooperation Ecological Regions of North America (Wiken et al., 2011): the Arctic Foothills, Seward Peninsula, and Brooks Range (Fig. 1). Across these regions, mean average temperatures range from −12 to −5 °C while mean annual precipitation ranges from less than 200 to 600 mm. This region's ecological setting and size, along with its well-documented history of vegetation and disturbance dynamics, make it ideal for benchmarking our mapping methods and testing the potential for application at continental scale.

**Fig. 1:**
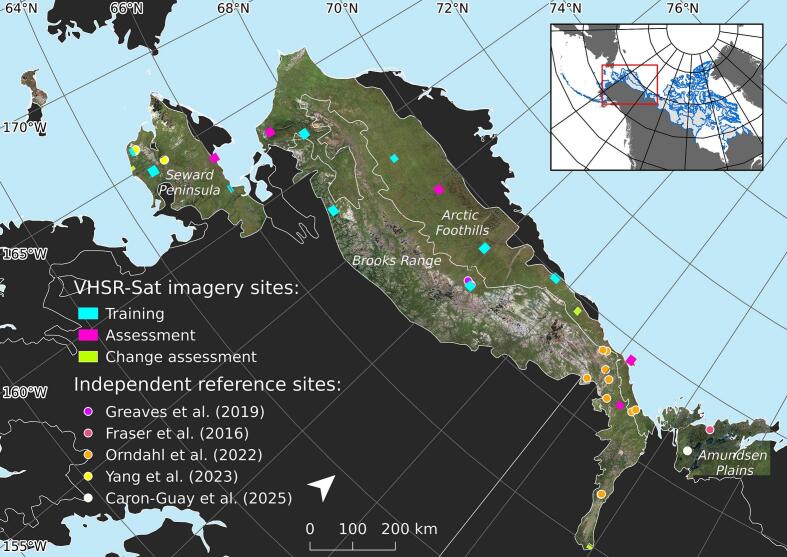
Map of true colour imagery from peak summer conditions in 2023, delineated by boundaries for the Arctic Foothills, Seward Peninsula, and Brooks Range ecoregions. The map also identifies sites with available very high spatial resolution satellite (VHSR-Sat) imagery used to train and assess mapping results (colored boxes) and independent sites with unmanned aerial vehicle (UAV) -derived reference maps used to assess fractional cover maps derived from Harmonized Landsat and Sentinel-2 (HLS; colored points). Part of the Amundsen Plains ecoregion, in the Mackenzie River Delta between Inuvik and Tuktoyaktuk, NT, Canada, is also included because two sites with independent reference data in this region were used for assessment. The inset map displays the study extent within the North American Arctic region. Independent reference site points are magnified for visibility; apparent spatial overlap with training sites does not indicate true co-location.

An important feature of this study area is its physiographic and ecological diversity, including alpine zones in the Brooks Range and British Mountains, the forest-tundra ecotone, hills and plateaus of the Arctic Foothills, and diverse coastal, upland, and montane regions in the Seward Peninsula. These ecoregions support heterogenous landscapes with shrubby hillslopes and riparian zones, moist tundra and wetlands dominated by sedges and mosses, tussock tundra, and dry uplands dominated by prostrate shrubs and lichens. Every tundra vegetation community unit from the Circumpolar Arctic Vegetation Map (CAVM; Walker et al., 2005) is found in this region.

Consistent spectral greening (Frost et al., 2025; Jia et al., 2003; Myers-Smith et al., 2020) has been widely documented in this region, and shrubification has been studied at field sites across the North Slope (Liljedahl et al., 2020; Naito and Cairns, 2015; Tape et al., 2012) and Seward Peninsula (Racine et al., 2004; Salmon et al., 2019; Schore et al., 2023; Yang et al., 2025a). The study area also has an extensive record of disturbance history. Both the Seward Peninsula and Noatak River Valley are hotspots for tundra fires (Hrobak et al., 2024), and permafrost disturbance has occurred throughout the domain in recent decades but is particularly common in eastern portions of the study region, on Qikiqtaruk (Herschel Island), and in the Peel Plateau (Nitze et al., 2025; Yang et al., 2025b).

In addition to the main study area, two sites from the Amundsen Plains were opportunistically included to support assessment of our mapping results. These sites are located along the Inuvik-Tuktoyaktuk Highway at Trail Valley Creek north of Inuvik, NT (68.7462° N, 133.5017° W) and near Tuktoyaktuk, NT (69.3658° N, 133.0350° W) in Canada, respectively, where independent very high-resolution reference maps allowed us to assess mapping results in an adjacent ecoregion that was not represented in the training data used to estimate our models.

## 3 Methods

We mapped sub-pixel land cover fractions annually from 2016 to 2023 using a framework that leverages the high temporal resolution (∼2 days) of the Version 2.0 HLS product with very high spatial resolution (∼2 m) site-level imagery from commercial satellites. Our methodology consists of four main steps (Fig. 2): 1) using the land surface phenology (LSP) algorithm developed by Bolton et al. (2020), synthetic spectral-temporal features corresponding to the timing of mid-green-up, peak greenness, and mid-green-down phenology at each pixel were estimated from HLS data; 2) training data were compiled by mapping land cover at 2-m spatial resolution from very high spatial resolution satellite (VHSR-Sat) imagery acquired during the HLS era (2016–2023), and then computing the sub-pixel fractional cover of each land cover type within 30-m HLS pixels based on these maps; 3) machine learning-based regression models were estimated to predict sub-pixel land cover fractions using spectral-temporal features derived from HLS in combination with a small set of ancillary features, and Monte Carlo resampling was used to estimate pixel-level uncertainty in the predicted fractional cover maps; and 4) the accuracy of fractional cover mapping results was assessed using five independent reference datasets from unmanned aerial vehicle (UAV) surveys, compiled from previously published studies. As part of this analysis, we also analyzed geographic patterns of uncertainty and quantified change across time in fractional cover maps, where the latter was assessed using repeat VHSR-Sat image pairs.

**Fig. 2:**
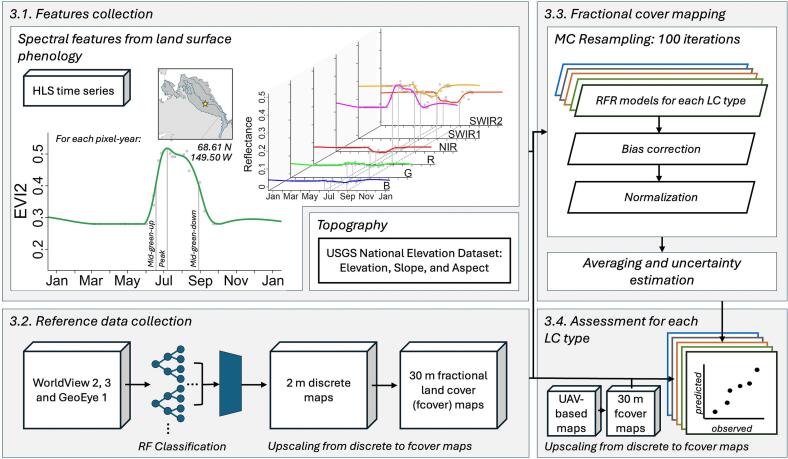
Schematic showing the framework used for mapping annual land cover (LC) fractions, quantifying model uncertainty, and assessing mapping results. Numbers in the upper left corner of each sub-panel correspond to subsections within the Methods section describing each step. Plots in the top-left panel (3.1. Features collection) illustrate how synthetic spectral features are extracted for dates corresponding to the timing of mid-green-up, peak two-band Enhanced Vegetation Index (EVI2), and mid-green-down for a representative pixel (identified by the yellow star). (For interpretation of the references to colour in this figure legend, the reader is referred to the web version of this article.)

### 3.1 Extracting spectral-temporal features using land surface phenology

Spectral-temporal predictors for each 30-m pixel were estimated using all available HLSS30 and HLSL30 surface reflectance imagery from January 2015 through December 2024. To estimate annual LSP at each pixel, we fit cubic smoothing splines to time series of the two-band Enhanced Vegetation Index (EVI2) using the Multi-Source Land Surface Phenology algorithm (MSLSP) described in Bolton et al. (2020). Synthetic spectra corresponding to key phenological dates at each pixel were estimated by fitting cubic smoothing splines to the time series for each spectral band and extracting modeled reflectance values for the dates corresponding to mid-green-up, peak EVI2, and mid-green-down at each pixel (Fig. 2).

Previous studies have demonstrated that differences in phenology can be used to differentiate land cover types using seasonal spectral-temporal metrics (He et al., 2019; Macander et al., 2017; Nill et al., 2022; Okujeni et al., 2021). At high latitudes, however, these metrics are prone to outliers because Arctic growing seasons are short (about two months, from mid-June to mid-August) and fixed seasonal periods may have few cloud-free images (Fig. S1). This challenge was substantial before the launch of Sentinel-2, and so efforts to map Arctic land cover fractions from Landsat imagery have been limited to using imagery spanning multiple years to produce time-averaged land cover maps (Beck et al., 2011; He et al., 2019; Macander et al., 2017) or time series of maps at multi-year (e.g., 5–6 year) intervals (Macander et al., 2022; Nill et al., 2022). Additionally, in the Arctic tundra, the start and end of the growing season can vary interannually by two or more weeks (Prevéy et al., 2022). Hence, fixed seasonal periods do not consistently capture phenological variation across years. By using spectral-temporal features aligned with LSP estimated from HLS, which provides a revisit frequency on the order of two days, our methodology captures representative spectra associated with specific land cover types and mixtures. Comparisons of interannual variability in LSP-derived synthetic spectra versus seasonal composites from HLS and Landsat at pixels with stable land cover shows significantly lower variability across years using our approach, with the largest differences in the NIR band, which is particularly important for vegetation mapping (Fig. S2). This indicates that our spectral-temporal features provide stable and reliable mapping inputs (for more information please see Section S1 of the Supplemental Information).

### 3.2 Training data collection from commercial satellite imagery

Our method for training data collection builds on previous work by Beck et al. (2011), who mapped shrub cover circa-2000 in the North Slope of Alaska using a combination of high spatial resolution SPOT and IKONOS imagery, and medium resolution Landsat data. Relative to 2011, the increased availability of VHSR-Sat imagery, paired with the higher temporal density of HLS data, enables scalable training of annual land cover models over large areas. For this work, we compiled training data using categorical land cover maps we produced from GeoEye 1 and WorldView 2 & 3 imagery at nine sites distributed across the study region (Fig. 1, Table S1). Sites were selected by targeting clear-sky, high-quality, peak-to-late growing season (typically early-July to late-August) images acquired between 2016 and 2023 with a minimum of two sites per ecoregion. To ensure geolocation accuracy, each image was orthorectified and co-registered to contemporaneous HLS imagery as detailed in Section S2 of the Supplemental Information.

Random forest classification models were estimated for each training site and used to classify imagery at 2-m resolution into seven classes: clouds, shadows, water, barren, low vegetation, shrub, and tree (Table 1). Classification models were trained using a minimum of 500 manually interpreted pixels per site, where each pixel was assigned to a land cover class if it comprised greater than 50% of the 2-m pixel. Input features included multispectral VHSR-Sat bands, and elevation, slope, and aspect derived from ArcticDEM (Porter et al., 2023). We used random forest classification (200 trees, minimum leaf size of 5) in an iterative procedure, where for each iteration, each map was assessed qualitatively and quantitatively, and training samples were added to underrepresented and frequently misclassified classes. To assess the accuracy of the high spatial resolution site maps, we computed overall accuracy as well as class-specific user's and producer's accuracies for each site following community established area-based accuracy assessment ‘good practices’ (Olofsson et al., 2014; Tyukavina et al., 2025). Overall classification accuracies for these site maps ranged from 91 to 97.3%. We provide details regarding the workflow, user's and producer's accuracies (Section S3), and confusion matrices reporting per-class accuracies for each site (Tables S2–S15) in the Supplemental Information. One training site (Site 8) was co-located with a UAV-derived independent reference map (Greaves et al., 2019), allowing us to compare this training map scaled to 30-m fractional cover with a high-quality independent dataset. Strong agreement with R^2^'s of 0.52–0.88, mean absolute errors (MAE) of 0.49–7.55% and average biases of −0.89–0.88% across land cover classes lend confidence to our ability to produce reliable training data from VHSR-Sat imagery (Table S16).

**Table 1:** Classes used for land cover mapping. Clouds and topographic shadows are masked out from the site maps.

Type	Class	Description
Mask	Clouds	Cloud and aerosol cover
	Shadows	Shadows from clouds or steep terrain and low solar illumination angles
Land cover	Water	Bodies of water including streams, lakes, ponds, and standing water in wetlands
	Barren	Non-vegetated land including developed areas (e.g., roads, pipelines, mines, and infrastructure)
	Low vegetation	Nonvascular, herbaceous, and dwarf shrub cover (e.g., *Vaccinium* spp., *Empetrum nigrum*, *Dryas* spp., *Cassiope tetragona*, and prostrate *Salix* spp.)
	Shrub	Low and tall shrubs (erect birch, willow, and alder)
	Tree	Coniferous trees (black and white spruce)

To compile training data for estimating models to perform sub-pixel fractional land cover mapping, we upscaled the 2-m classification results at each training site to create 30-m maps depicting the fractional cover for each class in each 30-m HLS pixel. 30-m pixels containing areas mapped as clouds or shadows in the 2-m classification were excluded from the training data.

### 3.3 Model estimation and bias correction

Thirty synthetic spectral features from HLS, augmented by three topographic features (Table S17) along with corresponding sub-pixel fractions from training samples, were used to estimate unique random forest regression models for each land cover class. Spectral features included HLS surface reflectance values for the six visible, near infrared, and shortwave infrared bands, EVI2, and the tasseled-cap greenness, brightness, and wetness indices (Baig et al., 2014) corresponding to the timing of mid-green-up, peak EVI2, and mid-green-down at each pixel. Topographic features included elevation, slope, and aspect from the 30-m USGS National Elevation Dataset (U.S. Geological Survey, 2019).

To select representative training pixels that capture variation in fractional cover at each training site, we used a stratified random sampling protocol targeting fractional shrub cover. Specifically, we divided the fractional shrub cover range (0–100%) into five equal-width bins (i.e., of 20%), and randomly sampled 1000 pixels from each training site, with a minimum of 10 pixels per bin. Only two sites had tree cover, and at these sites, to ensure that sparse tree cover was adequately represented in the training dataset, we instead stratified by tree cover using the same binning approach.

Regression models were estimated using the Random Forest algorithm (Breiman, 2001) implemented with the scikit-learn package (version 1.4.2) in Python 3.11.4. We used five-fold cross validation with Bayesian optimization (Snoek et al., 2012) implemented via the scikit-optimize package (version 0.10.2) to perform parameter optimization for our random forest models. Our models showed modest tendency to over-predict low fractional cover and under-predict high fractional cover (Fig. S3), a pattern commonly found in regression modeling and in previous studies (Huang et al., 2016; Macander et al., 2022; Nill et al., 2022; Stanimirova et al., 2022; Zhang and Lu, 2012). Following Huang et al. (2016), we attempted to correct this bias by applying a linear rotation to predictions using an independent set of 1000 samples from each training site that were collected for this purpose:

(1) $$\mathrm{Bias}=Y_{\mathrm{pred}}-Y_{\mathrm{obs}}=a+bY_{\mathrm{obs}}$$

This correction did not completely resolve the issue but improved overall accuracy and reduced bias in the uncorrected predictions (Fig. S3). Next, we normalized the predicted fractional cover in each land cover class in each pixel to sum to one. As a final postprocessing step, we spatially filled sparse no-data pixels occurring on mountaintops and coastal water bodies corresponding to areas where fractional cover could not be modeled due to lack of cloud-, snow- and ice-free observations from HLS for the mapping year. To do this we filled no-data pixels based on neighboring pixels and using the water occurrence layer from the Global Surface Water Dataset (Pekel et al., 2016) to identify pixels that intersect water bodies.

### 3.4 Accuracy assessment of fractional cover maps

We used two complementary approaches to evaluate the accuracy of the final 30-m fractional cover maps. First, we compared our mapping results against five independent datasets where land cover was mapped using UAV imagery (Fig. 1, Table S1; Caron-Guay et al., 2025; Fraser et al., 2016; Greaves et al., 2019; Orndahl et al., 2022; Yang et al., 2023). These data sources are fully independent and provide a rigorous basis for assessing our maps. Each reference map was resampled to 30-m resolution to enable comparison with our maps from HLS, and PFT class labels were cross-walked to match our land cover classes. Details of how we performed the cross-walking are found in Section S4 of the Supplemental Information along with a table showing each cross-walked class (Table S18). Second, we compared our fractional cover maps with five 30-m maps generated from commercial satellite imagery that were held out for assessment (Fig. 1, Table S1). A random sample of 1000 pixels was drawn from each assessment map for a total of 5000 samples. These comparisons provided an additional basis for assessment and demonstrate the utility of maps derived from commercial satellite imagery for model evaluation where UAV imagery is not available. In this context, it's important to note that VHSR-Sat assessment maps share the same modeling framework and data source as the training data, limiting their independence as a validation dataset; UAV-based assessments, by contrast, are fully independent datasets produced through separate field surveys.

To evaluate performance against results from previous studies, we compared our HLS-based maps and independent reference data from UAV-derived maps against three previously compiled fractional cover products that have spatial and temporal overlap with this study: 1) the 2015–2020 map for the greater Mackenzie Delta Region produced by Nill et al. (2022); 2) the map of Arctic and boreal Alaska and Yukon for 2020 produced by Macander et al. (2022); and 3) the circa-2015 map for the Alaska Tundra produced by He et al. (2019). We selected years in each case to match the central or closest year of each product. Because each map had different land cover class definitions, we cross-walked these maps to our mapping scheme. Further details describing how these comparisons and cross-walking (Table S19) were performed are included in Section S5 of the Supplemental Information, and limitations regarding cross-walking are discussed in the Discussion.

To investigate which input features contributed the most to the estimated models, we extracted feature importances from all model runs to quantify the contributions of different features for each land cover type. Additionally, we plotted the feature values of pure endmembers with 100% cover in each class across training sites to examine land cover separability across input features.

### 3.5 Quantifying pixel-level uncertainty

Pixel-scale, sampling-based modeling uncertainty associated with our fractional cover predictions was quantified using a Monte-Carlo resampling approach. To do this, we extracted 100 independent training datasets for each of the nine training site maps, which we then used to estimate 100 independent random forest models for each land cover type, from which we computed the mean predicted fractional land cover along with the associated standard error for each class for each pixel-year. The standard error was computed before post-processing, so that no-data areas are included in the uncertainty product, allowing users to identify pixels that were not modeled but gap-filled.

The spatial distribution of uncertainty in modeled pixel-level fractional land cover was analyzed for 2020, a central year in our time series that is representative of uncertainties across all years (Table S20). We examined how uncertainty varied across the range of land cover fractions by comparing them with the frequency distribution of pixel-level fractional cover values for each land cover type. We also evaluated the realism of estimated pixel-scale uncertainties by exploring the relationship between uncertainty and prediction errors using data from the five independent reference sites with high-resolution UAV maps.

### 3.6 Characterizing and assessing change through time

A key goal of our methods is to provide a robust foundation for mapping land cover change, including abrupt changes from disturbance, post-disturbance succession, and gradual transitions in composition (e.g., shrubification). To evaluate the ability of our method to capture abrupt changes related to disturbance events, we examined time series of modeled annual land cover fractions at sites with known fire and permafrost disturbance perimeters. Specifically, we used the Alaska Large Fire Database (Hrobak et al., 2024) and the Database of AI-detected Arctic retrogressive thaw slump (RTS) and active layer displacement (ALD) footprints (DARTS; Nitze et al., 2025) to locate three fire and two permafrost disturbance perimeters, respectively. Two additional RTS events at change assessment site 3, which were undetected in DARTS, provided additional change evaluation sites in the Peel Plateau region of the Brooks Range (Fig. 1, Table S1).

To evaluate the ability of our methods to capture continuous land cover change, we compared change inferred from our maps with change observed from repeat VHSR-Sat imagery. To quantify gradual change, previous studies have fit linear models to times series of fractional cover to map long-term trends (Lewińska et al., 2023; Nill et al., 2022) or tested the significance of total change across time (Macander et al., 2022). Because this study relied on HLS time series and was therefore limited to eight years (2016–2023), we used the Theil–Sen slope estimator in combination with the Mann–Kendall significance test (Sen, 1968; Theil, 1992) to estimate monotonic trends in pixel-level fractional cover. While the short study period limits our ability to robustly characterize long-term trends, we expected to still detect changes in fractional cover in areas with consistent and directional land cover transitions. We did not explicitly model structure breaks from disturbance events due to the difficulty of reliably identifying breakpoints over short time series.

We compared modeled change with observed change using repeat VHSR-Sat image pairs collected from each of the three ecoregions in the study area (Fig. 1, Table S1). All images were orthorectified and co-registered to HLS imagery and to each other, following the procedure described in Section S2 of the Supplemental Information. For each image pair, we selected 60 pixels, evenly split between pixels that exhibited statistically significant increases in fractional cover, statistically significant decreases in fractional cover, and non-significant change for each of the land cover classes excluding the tree class, resulting in a total of 720 assessment pixels. Trees were excluded as they are much slower growing and make up a very low proportion (< 2% mapped land cover) of our study area. Fractional land cover changes were manually interpreted within each 30-m assessment pixel for observed change estimates.

## 4 Results

### 4.1 Accuracy of mapped land cover fractions

Across the study domain, annual maps of fractional land cover predicted by our models are consistent with expected biogeographic patterns in vegetation and land cover (Fig. 3). Notable large-scale biogeographic patterns include thermokarst lakes on the North Slope, a dense network of lakes and meandering rivers in the Mackenzie Delta (Fig. S4a), barren mountaintops in the Brooks Range and British Mountains (Fig. S4b), the northern treeline at the tundra-taiga ecotone along the southern Brooks Range ecoregion (Fig. S4e), a latitudinal gradient in shrub cover from shrublands in the southern Brooks Range ecoregion to tussock-dominated foothills on the North Slope, and extensive areas with high shrub cover in the Mackenzie Delta and southern Seward Peninsula (Fig. 3). Results also capture the prevalence of shrubs along riparian zones and hill slopes, as highlighted in visual agreement between high resolution imagery, reference maps, and predicted shrub cover at training, assessment, and independent reference sites (Fig. 3a – c).

**Fig. 3:**
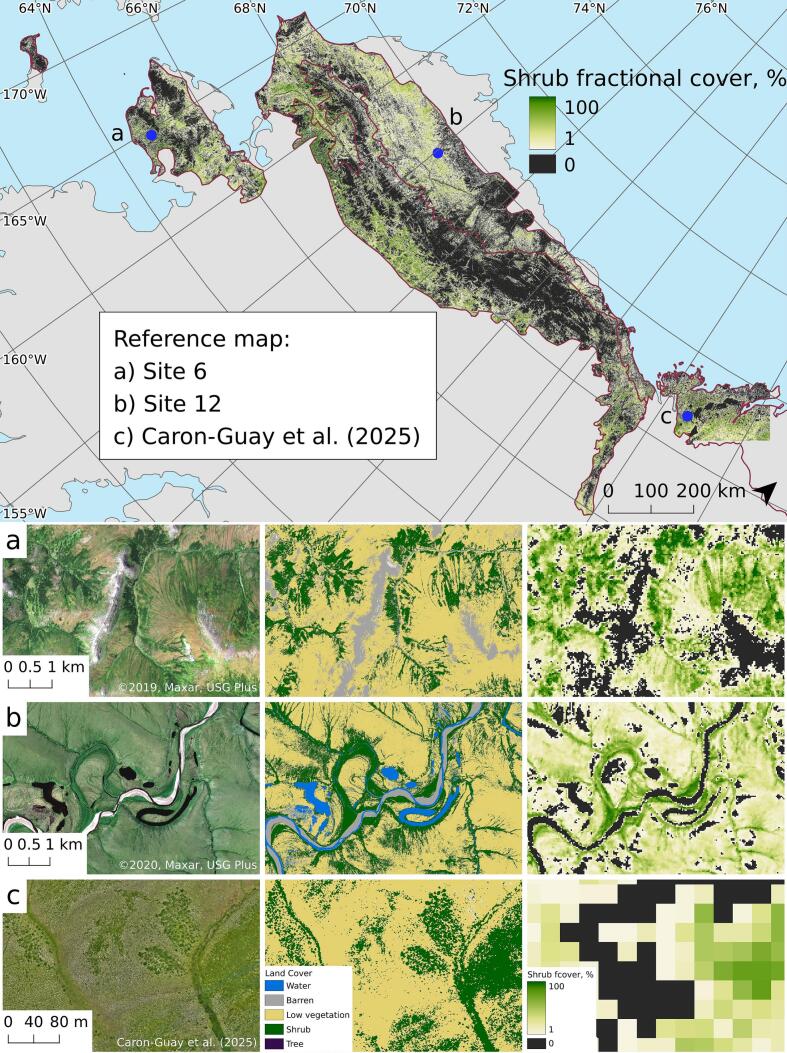
Mapped shrub fractional cover is consistent with expected biogeographic patterns and reference data. Shrub fractions are shown here for 2020 for the entire study region (map) along with results for one site used for training data (a), one assessment site (b), and one site with independent reference data derived from unmanned aerial vehicle (UAV) imagery (c). The location of each site is shown on the map as a labeled blue dot. For each site we show the true colour image on the left, classification results at ≤2 m in the middle, and model-predicted shrub fractional cover (fcover) at 30 m for the corresponding data year on the right. (For interpretation of the references to colour in this figure legend, the reader is referred to the web version of this article.)

Assessment of fractional cover maps against independent reference data derived from UAV imagery reveals strong overall agreement across all four tundra land cover types with R^2^ values of 0.42–0.77, MAEs of 0.69–15.98%, and average biases of −0.88–0.81% (Fig. 4). The low average bias (magnitudes <1% across all land cover types) and MAE values provide confidence that sub-pixel land cover composition predicted by our methods are robust and reliable. Accuracies were highest for non-vegetated classes (water and barren cover), although models tended to modestly underpredict fractional cover in these classes (Fig. 4a – b). For vegetated tundra classes, low vegetation cover was overpredicted at lower fractional cover (∼ < 80%) and shrubs were underpredicted at higher cover fractions (∼ > 20%, Fig. 4d – e).

**Fig. 4:**
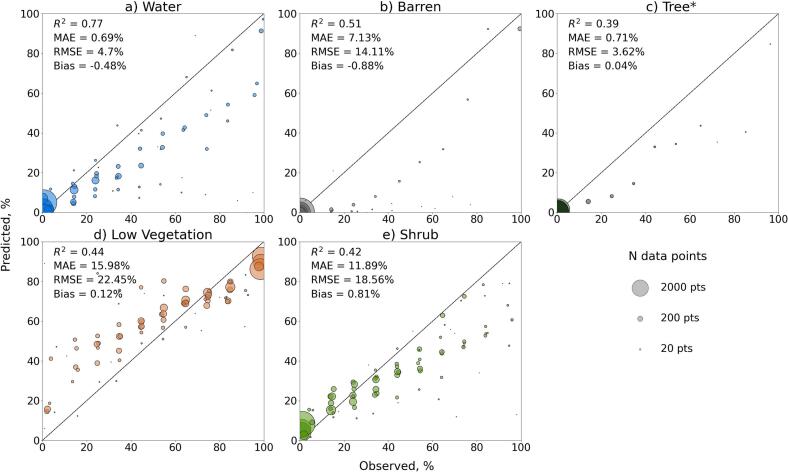
Assessment of predicted against observed fractional cover for (a) water, (b) barren, (c) tree, (d) low vegetation, and (e) shrub cover. Each circle represents a bin where observed cover at each independent reference map has been aggregated into 10% fractional cover bins from 0 to 100% for each site. The radius of each circle corresponds to the number of data points (pixels). Regression statistics were calculated using all data points. For tundra land cover types, observations were from unmanned aerial vehicle (UAV) -based maps at independent reference sites. *For the tree class, observations were from VHSR-Sat assessment sites, as there was insufficient tree cover at the independent reference sites.

Mapped tree cover could not be robustly assessed at independent reference sites due to limited data availability. Only one dataset (Orndahl et al., 2022) had any mapped trees, with just 12 pixels containing tree cover, all with low cover fractions (< 15%). That said, accuracy assessment at a VHSR-Sat assessment site that had a larger range of mapped tree cover (0–96%) demonstrated good agreement between modeled and independent reference data, with an R^2^ of 0.39 and low MAE (0.71%) and bias (0.04%; Fig. 4c, Fig. S5). For other land cover types, assessment against VHSR-Sat assessment sites yielded MAEs that were consistent with those obtained from UAV-based maps (within 2%, Fig. S5).

Comparisons of results from this study with fractional cover mapped in previous studies show that our maps agree most closely with results from Nill et al. (2022), with higher R^2^'s, and lower disagreement and biases compared to other products across most land cover classes (Fig. 5, S6 – S8). Additionally, while this study captures a full range of shrub fractional cover from 0 to 100%, the Nill et al. (2022) product estimates many cases of high shrub fractional cover where this study maps no shrub cover, the Macander et al. (2022) product predicts few pixels with over 70% shrub cover and no shrub cover in pixels where our maps predict a wide range of shrub cover, and the He et al. (2019) product similarly captures a narrow range of shrub fractional cover (approximately 10–60%) where our predictions span 0–100% (Fig. 5).

**Fig. 5:**
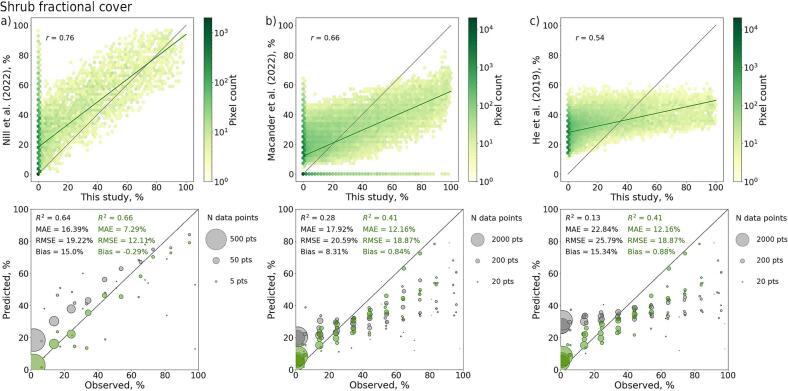
Plots comparing shrub cover fractions predicted in this study against independent map products for the same time period (top), and assessments against unmanned aerial vehicle (UAV) -based maps at independent reference sites with assessment points and statistics for this study in green and for the other map products in gray (bottom). (For interpretation of the references to colour in this figure legend, the reader is referred to the web version of this article.)

Evaluated against independent reference data, the Nill et al. (2022) maps tended to overpredict at low fractional cover across all land cover types, while maps from Macander et al. (2022) and He et al. (2019) showed a strong tendency toward regression to the mean relative to results from this study (Fig. 5, S6 – S8). Overprediction of low fractional shrub cover in these products may arise from different class definitions as opposed to reflecting valid differences between the maps, as the cross-walked shrub classes (Table S19) in Macander et al. (2022) and He et al. (2019) include deciduous dwarf shrubs and evergreen dwarf shrubs, respectively, while the reference data treats all dwarf shrubs as low vegetation. However, these definitional mismatches do not account for the consistent underprediction in locations with high fractional shrub cover.

### 4.2 Spectral-temporal separability of continuous land cover

Relative importance plots derived from random forest show that the importance of individual LSP-derived spectral-temporal features varied by land cover type (Fig. 6). For vegetated land cover classes, spectral information corresponding to the date of peak greenness was most important for predicting sub-pixel cover fractions (Fig. 6c, d). The red band and EVI2 were particularly important for distinguishing between shrubs and low vegetation endmembers during peak greenness (Fig. 7). Features extracted for dates corresponding to the timing of mid-green-up and mid-green-down (henceforth, shoulder-season features) were also informative, contributing on average about 10% and 4% of the total normalized feature importances for low vegetation and shrub fraction models, respectively (Fig. 6c, d).

**Fig. 6:**
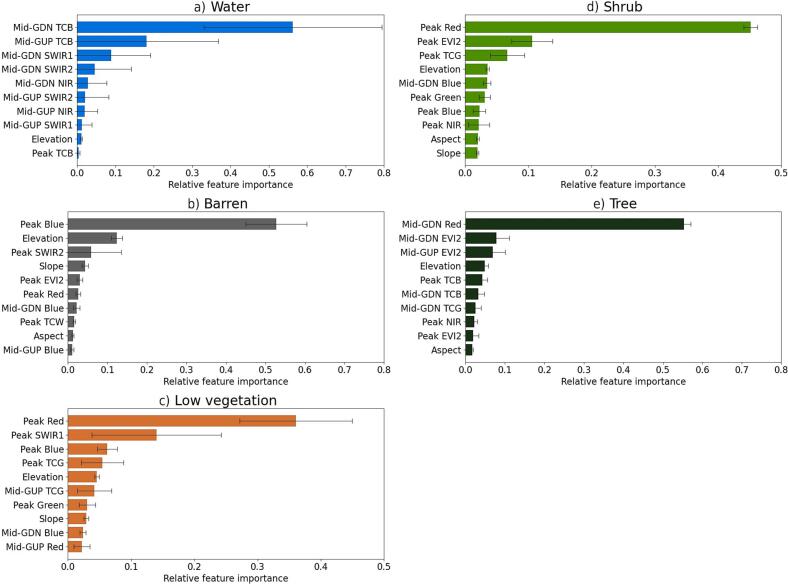
Relative importance plots showing the ten most important features for each land cover type. Error bars represent standard deviations from the 100 model runs. Mid-GUP and Mid-GDN refer to mid-green-up and mid-green-down, respectively. (For interpretation of the references to colour in this figure legend, the reader is referred to the web version of this article.)

**Fig. 7:**
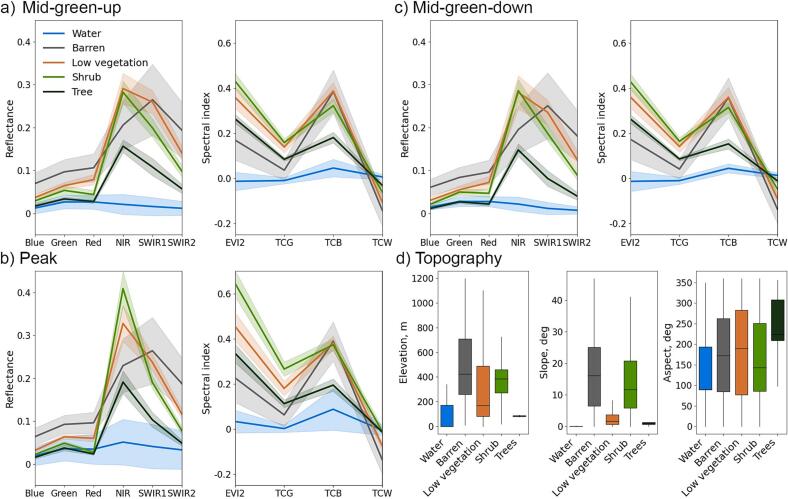
Reflectance spectra and vegetation indices (shaded envelopes showing ±1 standard deviation) for key points in the two-band Enhanced Vegetation Index (EVI2) phenological curve (a, b, and c) and topographic distribution (d) for all 30-m endmember pixels for each land cover type across all training site.

Shoulder-season features were most important for predicting the fractional area of water and tree cover (Fig. 6a, e). For water, endmember plots show greater spectral variability during peak greenness, which may be caused by increased sediment flow and algal/vegetative growth across water bodies and channels in the summer (Fig. 7). This makes shoulder-season features more reliable for predicting water cover. For trees, because the dominant trees at the taiga-tundra ecotone are evergreen, spectral differentiation against predominantly deciduous tundra vegetation occurs in the shoulder seasons when most tundra vegetation has senesced.

Boxplots showing the distribution of low vegetation and shrubs as a function of topography clearly show that landscape position is an important control on vegetation composition. Shrubs are more prevalent on steeper slopes and at modestly higher elevations (Fig. 7d), which tend to be well-drained and underlain with ice-poor permafrost (Gaglioti et al., 2021). These topographic controls are captured by the random forest models, as elevation and slope are among the top ten predictors for low vegetation and shrub fractions (Fig. 6c, d).

### 4.3 Spatial patterns of uncertainty

To investigate spatial patterns of model uncertainty, we mapped and analyzed the standard deviation in model predictions at each pixel across 100 models estimated via Monte Carlo resampling of the training data for the year 2020 (Fig. 8). Overall, pixel-scale uncertainty in predictions was low, with median values of 0–4.24%. To investigate if and how uncertainty depends on fractional cover, we computed the average standard deviation in 10% land cover fraction bins for each cover type. Overall, uncertainty tends to be higher in pixels with mixed or heterogenous land cover composition (i.e., fractional cover between 10% and 90%). However, apart from the low vegetation class, which had the most bimodal distribution with peaks at both high and low fractional cover, the distribution of fractional cover is highly skewed toward low fractional covers (< 10%), with lower uncertainty in predictions.

**Fig. 8:**
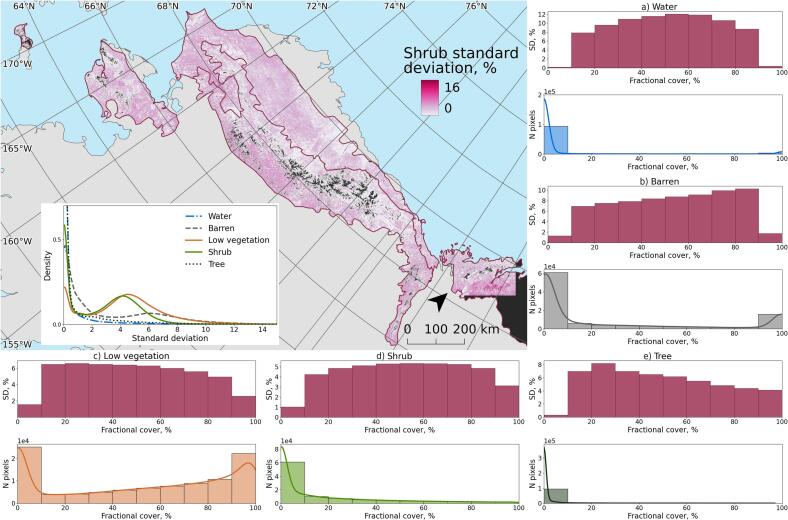
Distributions of uncertainty in fractional shrub cover for the year 2020 from Monte Carlo uncertainty estimation (map) with paired histograms show that uncertainty is lower for pixels with fractional cover ranges that occur more frequently across the study area (a – e). Histograms show the median uncertainty and frequency distribution of fractional cover in 10% bins for each land cover type. The inset plot on the map shows the frequency distribution of standard deviation in predicted fractional cover for each land cover type across 100 model predictions. Areas in black on the map represent no data and correspond to pixels with no clear, snow- and ice-free observations for the mapping year, which were mostly located in alpine zones with very low vegetation cover.

To further evaluate the representativeness with which model uncertainties characterize errors in model predictions, we plotted the magnitude of prediction errors from independent assessments against UAV-derived reference data as a function of model-derived uncertainties (Fig. 9). Results show that the average magnitude and variance of prediction errors increase monotonically as a function of model uncertainty, which demonstrates that the estimated model-based uncertainties capture information related to model accuracy.

**Fig. 9:**
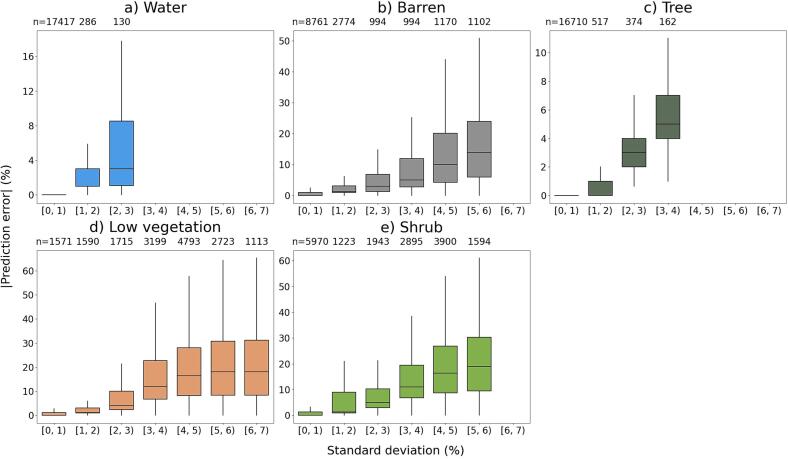
Prediction error magnitude (from comparisons with independent reference data) increases with model uncertainty (standard deviation in model predictions across 100 Monte Carlo predictions) across land cover types. n is the number of pixels per bin. For plots (b), (d), and (e), boxplots are only shown for bins that contain >5% of all 30-m pixels at the independent reference sites. However, for land cover classes that are underrepresented (Water and Tree) bins containing >100 pixels are included.

### 4.4 Land cover change

We evaluated the effectiveness of our mapping results for identifying and quantifying both abrupt change from disturbance events and long-term change including slow and continuous changes (e.g., shrubification). Annual maps in locations with fire disturbances show sharp increases in barren cover along with loss of vegetation cover in the year directly following fire events (Fig. 10). Mapping results also captured unburned islands located within fire perimeters and heterogeneity in fractional barren cover across burn scars that are highly correlated with burn severity (Fig. S9). Post-fire decreases in barren cover with concomitant increases in low vegetation and shrub cover reflect rapid post-fire recovery of vegetation, with low vegetation approaching pre-fire levels within three years.

**Fig. 10:**
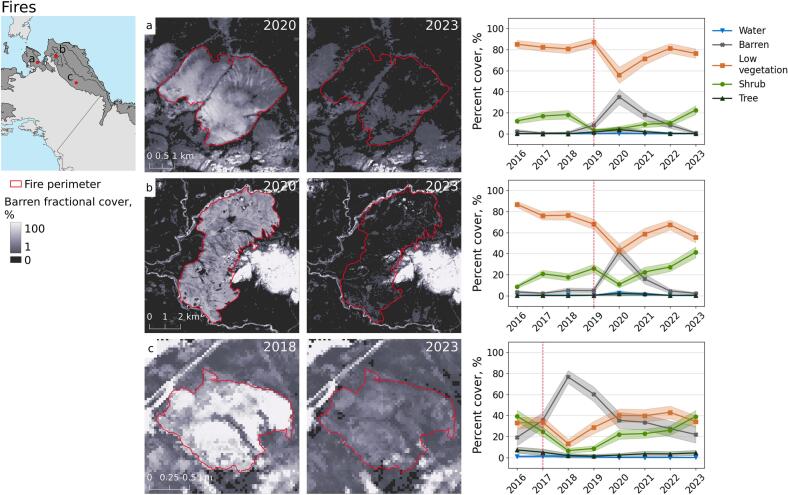
Model results capture vegetation loss and recovery from fire on an annual timestep. Maps show post-disturbance barren fractional cover for the year following fire and 2023, along with annual time series, for the 2019 Kugruk River fire #1 (a), 2019 Kaluktavik River fire (b), and 2017 Dietrich River fire (c). Fire perimeters are from the Alaska Large Fire Database (Hrobak et al., 2024). Shaded envelopes in the time series plots correspond to average uncertainty in mapped fractional cover over the disturbed area, and the dashed red line indicates the fire year. (For interpretation of the references to colour in this figure legend, the reader is referred to the web version of this article.)

Time series of land cover fractions also capture the gradual expansion of retrogressive thaw slumps, wherein maps of barren cover in 2018 and 2023 align well with detected slump perimeters from DARTS (Fig. 11a, b). Additionally, the time series of the coastal slump events on Hershel Island (Qiqiktaruk) shows evidence of coastal erosion inside of retrogressive thaw slump perimeters, as evidenced by the increase in fractional water cover (Fig. 11a). Our maps also identified land cover changes arising from two slump events that are not captured in the DARTS dataset but were present at change assessment site 3 (Table S1) in the Peel Plateau region of the Brooks Range. These changes are located in the taiga-tundra ecotone, and both RTS time series display loss of vegetation, particularly trees, to barren cover (Fig. 11c, d).

**Fig. 11:**
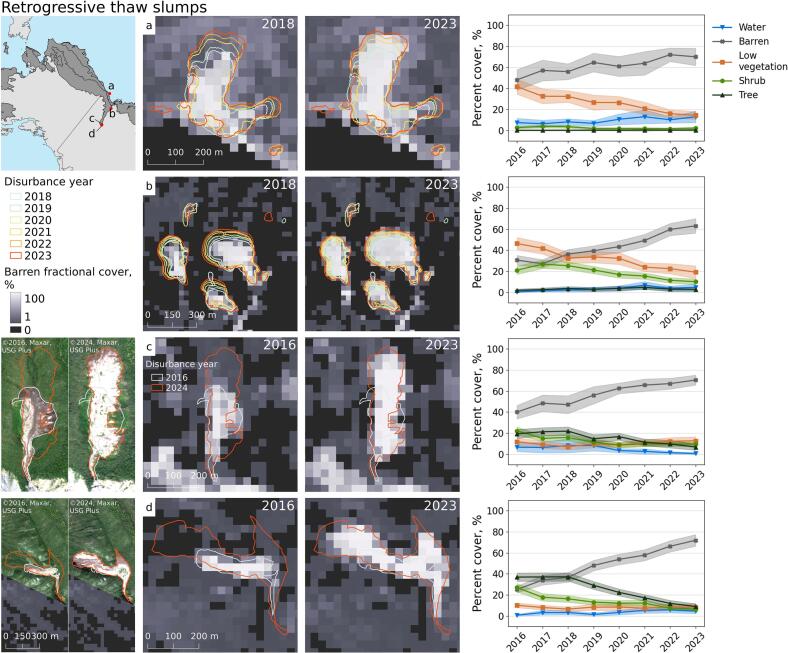
Annual estimates of land cover change capture retrogressive thaw slump (RTS) expansion within known (a, b) and previously unrecorded (c, d) RTS perimeters. Maps show barren fractional cover for the first recorded year of disturbance and 2023, and annual time series detail land cover change within all perimeters on the coast of Qikiqtaruk (a), and in the Peel Plateau (b – d). RTS perimeters for (a) and (b) are from DARTS (Nitze et al., 2025) while perimeters for (c) and (d) were interpreted from repeat very high spatial resolution (VHSR-Sat) images (true colour shown on the left) at change assessment site 3 (Table S1). Shaded envelopes in the time series plots correspond to average standard deviations over the disturbed area.

At change assessment sites, model-based estimates of trends in pixel-level fractional cover showed reasonable statistical agreement (R^2^ = 0.43–0.55; mean bias of −4.63–1.19%) across land cover classes but also tended to over-estimate changes with ratios of observed to predicted change that ranged from 0.39 to 0.81 (Fig. 12). Categorizing detected changes into three bins corresponding to increasing, stable, and decreasing fractional cover in each class, we found high categorical accuracies for low vegetation and shrub (76.67% and 77.78%) and only marginally lower accuracies for barren and water cover (73.33 and 75%). These results suggest that while the magnitudes of mapped changes are biased, the model-based results successfully capture the direction of land cover change at 30 m.

**Fig. 12:**
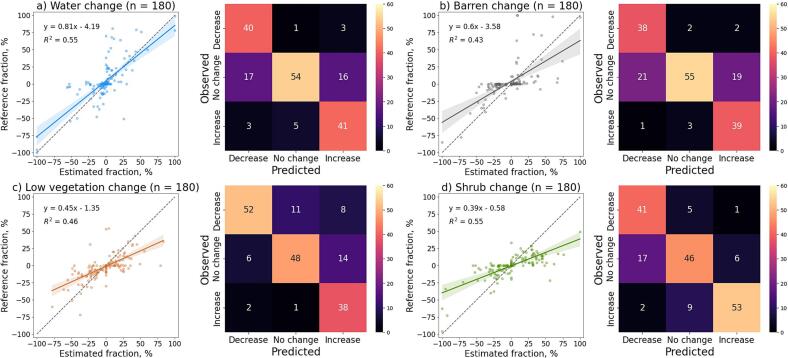
Assessment of predicted fractional cover changes against observed fractional cover changes from three very high spatial resolution satellite (VHSR-Sat) change assessment sites (Fig. 1, Table S1) for (a) water, (b) barren, (c) low vegetation, and (d) shrub cover. Regression plots show observed change data at VHSR-Sat change assessment sites against map-estimated change using the Theil-Sen slope, evaluating agreement in change direction and magnitude. Confusion matrices show the same results binned into direction of change to evaluate agreement in change direction.

Visual inspection of modeled changes in barren, water, and shrub cover at the three change assessment sites shows that mapped trends correspond well with observed changes in high resolution imagery. For example, our maps captured the expansion of barren ground due to mining near Nome, Alaska, shifts in water cover from braided river migration on the Alaskan North Slope, and shrubification along riverbanks in the Peel Plateau region (Fig. S12).

## 5 Discussion

This study establishes a novel framework for mapping sub-pixel land cover fractions in Arctic tundra, combining spectral-temporal features associated with phenological timing and high spatial resolution reference data from commercial satellite imagery. By aligning spectral features with biologically meaningful points in each pixel's phenological cycle, we capture vegetation dynamics with greater ecological relevance and temporal consistency than conventional compositing or harmonic modeling approaches (Fig. S2). Using random forest regression models trained on these data, we generated the first annual, wall-to-wall maps of sub-30-m mixtures of water, barren, low vegetation, shrub, and tree cover across the Arctic Foothills, Seward Peninsula, and Brooks Range ecoregions of Alaska (Fig. 3). Our maps demonstrate robust performance relative to existing static and multitemporal map products (Fig. 4, Fig. 5, S6 – S8) and quantify prediction uncertainty at the pixel level, enabling spatially explicit, pixel-level assessments of confidence in mapped outputs (Fig. 8, Fig. 9), which is especially critical in Arctic landscapes where field-based validation is sparse and surface heterogeneity is high. Results also successfully capture abrupt vegetation change from disturbance (Fig. 10, Fig. 11), and using repeat VHSR-Sat image pairs, we provide the first evaluation of gradual vegetation change trends arising from rapid climate change at high latitudes (Fig. 12, S12). This mapping approach offers new opportunities to monitor land cover dynamics at spatial and temporal resolutions that better reflect the fine-scale heterogeneity of ecological change occurring across the Arctic tundra. The resulting maps provide a valuable foundation for long-term monitoring of vegetation dynamics arising from climate change and disturbance across the North American Arctic.

### 5.1 Spectral-temporal features from land surface phenology improve fractional land cover predictions

We address the challenge of separating sub-pixel mixtures of spectrally similar tundra vegetation types by leveraging spectral-temporal features that are anchored to the phenological cycle at each pixel in each year (Fig. 2). This LSP-informed approach ensures that spectral-temporal features are ecologically grounded, capturing more consistently the timing of mid-green-up, peak greenness, and mid-green-down relative to methods based on annual composites or harmonic models. As a result, the spectral-temporal features used in this study show low variability across years for pixels with stable land cover and therefore provide a strong foundation for distinguishing land cover compositions (Fig. S2). While it is well-established that phenological differences can help distinguish spectrally similar vegetation types in Arctic tundra (Beamish et al., 2017; He et al., 2019; Macander et al., 2017), previous fractional cover mapping studies in the Arctic using temporally sparse Landsat time series (Zhang et al., 2022) have relied on multi-year fixed-period seasonal composites (Beck et al., 2011; He et al., 2019; Macander et al., 2017; Nill et al., 2022) or harmonic models derived from multi-year fits (Macander et al., 2022). While these methods leverage seasonality, they do not account for interannual variability in phenological timing, meaning features do not reflect consistent periods in a pixel's phenological cycle across years. By using temporally-dense HLS time series and a state-of-the-art LSP algorithm (Bolton et al., 2020), our methods synthesize annual spectral-temporal features associated with phenological timing at each pixel, providing a more reliable basis for predicting land cover fractions.

### 5.2 VHSR-Sat imagery enables robust, scalable model training and assessment

Reference data sampled from ∼2-m VHSR-sat imagery address long-standing challenges involved in acquiring representative high-quality ground truth data in support of Arctic tundra land cover mapping. Specifically, the dramatic increase in available high spatial resolution commercial satellite data over the last decade enables land cover to be classified at high spatial resolution over areas that are difficult to access and sample in the field (Beck et al., 2011). While not a replacement for field-based or UAV-based vegetation surveys, VHSR-sat imagery provides geographically extensive (Fig. 1) and high quality (table S16) data for training and assessing fractional land cover models in the Arctic. This improves representativeness compared to field-based vegetation survey data, which are spatially clustered and temporally restricted in the Arctic (Beamish et al., 2020). Because sub-10 m classification maps from VHSR-sat imagery can be upscaled to provide fractional land cover at coarser spatial resolutions, they can also provide more suitable, adaptable reference data that complement field survey data, which often have inconsistent plot geometry and geolocation error, and are generally collected at spatial scales (frequently 1 m^2^) that do not match the spatial resolution of medium resolution remote sensing datasets (Berner et al., 2024; Steckler et al., 2025; Zhu et al., 2024). When used to train models for fractional land cover, field data with spatial clustering and plot-to-pixel scale mismatches can exacerbate regression-to-the-mean and lead to poor transferability (He et al., 2019) when evaluated against independent UAV-derived reference data (fig. S7, S8)

One solution that has been used to overcome scarcity in high quality training data is to generate synthetic training data via linear mixing of pure endmember spectra across the range of possible mixtures of land cover classes (Nill et al., 2022; Okujeni et al., 2021; Stanimirova et al., 2022). However, this method relies on accurate identification of endmember pixels and the assumption that spectral features scale linearly across mixtures. In reality, homogeneous patches of vegetation >900 m^2^ are rare in the tundra, and light interactions among surface objects can be nonlinear (Borel and Gerstl, 1994; Keshava and Mustard, 2002). By leveraging VHSR-Sat imagery, our models are trained on representative mixtures of land cover that capture realistic, nonlinear scaling and variability in surface reflectance across heterogeneous Arctic land cover.

### 5.3 Improvements in mapping realism and quantification of uncertainty

Our annual maps showed good agreement, with R^2^ values of 0.42–0.77, MAEs of 0.69–15.98%, and average biases of −0.88–0.81% across land cover types, when compared against independent UAV-derived reference data (Fig. 4). Although these UAV-derived maps have high (but modestly variable) accuracy due to differences in classification algorithms and training inputs, results were consistent with comparisons against VHRS-Sat assessment maps (Fig. S5), which, while less independent, were obtained using a methodologically consistent modeling framework (Fig. S21), lending confidence to our accuracy estimates. These accuracies were comparable to or exceeded previous mapping efforts (Beck et al., 2011; He et al., 2019; Macander et al., 2017; Macander et al., 2022; Nill et al., 2022). Further, comparisons with map products from Nill et al. (2022), Macander et al. (2022), and He et al. (2019) revealed that our maps showed substantially stronger correspondence with independent UAV-derived reference datasets (Fig. 5; Fig. S6 – S8).

In addition to improved spectral-temporal predictors from HLS and high-quality training data from VHSR-Sat imagery, our maps likely also benefitted from the empirical bias correction step (Section 3.3, Eq. 1). Bias correction has been adopted in similar fractional cover mapping contexts in other regions (Huang et al., 2016; Stanimirova et al., 2022), but previous fractional cover products for the Arctic do not incorporate this type of correction. In our comparisons, existing maps, particularly those trained on field survey data, estimated a narrower range of fractional cover values for each vegetation type when compared to this study (Fig. 5, Fig. S6 – S8), which is a characteristic of machine learning-based regression models. When compared against independent reference data, our results show less-pronounced regression-to-the-mean and smaller biases at both low and high fractional cover relative to observed values (Fig. S6 – S8). While systematic overestimation of shrubs in areas with low shrub cover in the Macander et al. (2022) and He et al. (2019) data products can be partly explained by differences in land cover class definitions, underestimation of high shrub cover and overestimation of sparse low vegetation is attributable to prediction error. Specifically, the shrub class in Macander et al. (2022) and He et al. (2019) include all deciduous shrubs and all woody vegetation, respectively, inflating shrub estimates across the full range of fractional cover values compared to the reference data. However, in our evaluation, both products underestimated shrub cover and overestimated low vegetation where shrubs are most dense. Our results demonstrate that bias correction can improve fractional land cover estimation and is an important modeling step for mapping fractional vegetation cover in the Arctic.

Our methods also improve utility and interpretation of map products by providing pixel-level estimates of mapping uncertainty (Fig. 8). Comparisons of model uncertainties with the magnitudes of prediction errors at independent reference sites revealed a monotonic and positive relationship between uncertainty and prediction error across all land cover types, demonstrating that our uncertainty maps capture meaningful information related to the quality of predicted land cover fractions at each pixel (Fig. 9). Uncertainty estimates enable end users to evaluate quality and propagate uncertainty into downstream modeling analyses. In the past, fractional land cover products largely have not provided pixel-level estimates of mapping uncertainty (Baumann et al., 2018; He et al., 2019; Macander et al., 2017; Nill et al., 2022; Schug et al., 2024). This limits the utility of these products as prediction errors from regression mapping can be spatially structured and obscure signals of change (Goetz et al., 2021). In this study, we used Monte Carlo resampling to quantify sampling-based modeling uncertainty, an approach that has been previously used to map uncertainty in other variables such as above-ground biomass (Berner et al., 2018; Orndahl et al., 2025; Wang et al., 2021). However, challenges remain regarding optimal propagation of uncertainties arising from uncertainties in spectral-temporal features (e.g., due to atmospheric and topographic correction or LSP model fits) and errors in reference data (e.g., mislabeling or co-registration inaccuracies) (Macander et al., 2022). Further, while aggregating reference data to 30 m fractional cover reduces the impact of individual high-resolution pixel mislabeling through spatial averaging, spatially autocorrelated errors can still lead to biases in the upscaled data used to train our models. These sources of error and uncertainty are implicitly captured in our methods for quantifying uncertainty, but comprehensive attribution and propagation of errors are difficult and were not provided in this analysis.

### 5.4 Annual maps capture abrupt and gradual land cover change

Land cover maps at annual timestep capture changes in low vegetation and shrub recovery in the first three years after disturbance from fire (Fig. 10), findings that are consistent with results from field-based studies of post-fire successional processes in Arctic tundra (Frost et al., 2020; Phoenix et al., 2025). Our results also capture spatial patterns related to burn severity and unburned islands (Fig. S9), which can lead to local variability in early successional processes and introduce heterogeneity in the long-term recovery of Arctic vegetation (Chen and Lantz, 2024). Additionally, our mapping results identified annual expansion in previously identified RTS perimeters (Nitze et al., 2025) and successfully resolved additional, previously unrecorded cases of RTS events (Fig. 11).

Previous fractional cover mapping efforts, constrained by low frequency cloud-free observations during the Arctic growing season (Berner et al., 2020), have used multitemporal image composites that span multiple years for mapping. As a result, short-term dynamics or changes arising from pulse events (i.e., wildfires, abrupt permafrost thaw) are not captured in map outputs (Nill et al., 2022). This is evident in known fire perimeters across 2015–2020 in the map products from Macander et al. (2022) (Fig. S10), which do not capture short-term processes of fire and recovery that occur between map years. While both existing multi-year products appear to capture incremental disturbance processes from RTS expansion, our maps predict greater magnitudes of change and can more finely resolve annual slump areas (Fig. S11). By improving the temporal resolution at which land cover change can be estimated, our maps address a growing need for improved monitoring and attribution of land cover change dynamics associated with the intensification of wildfires (Descals et al., 2022; Hanes et al., 2019; Hu et al., 2015; Li et al., 2024) and abrupt permafrost thaw in the Arctic (Barth et al., 2025; Chen et al., 2021b; Foster et al., 2022; Natali et al., 2021; Segal et al., 2016).

Shrub expansion is the most widely documented vegetation composition change in the Arctic associated with climate warming, with evidence from repeat photography, long-term field observations, and remote sensing spanning multiple decades (García Criado et al., 2025; Elmendorf et al., 2012; Forbes et al., 2010; Fraser et al., 2014; Tape et al., 2012; Mekonnen et al., 2021). In this context, the annual fractional cover maps presented here offer a promising foundation for monitoring this trend. Our study provides the first known validation of mapped fractional land cover change in the Arctic (Fig. 12) and offers a new approach for acquiring reference data characterizing gradual transitions in tundra vegetation where repeat field surveys are often not feasible. Although this study spans only eight years, we were able to successfully map changes in the fractional cover across each tundra land cover class (Fig. 12, S12). With additional years of data, the signal-to-noise ratio available to identify and map change will improve, enabling more robust detection of long-term trends. Comparison of mapped change against reference data at VHSR-Sat change assessment sites revealed high accuracies in the direction of change but systematic overestimation of land cover changes (Fig. 12) that are consistent with biases noted in fractional land cover change studies in other biomes (Stanimirova et al., 2022), underscoring the need for high quality reference data that document changes in Arctic land cover. While previous studies have used Landsat to quantify long-term shrub expansion at regional scale (Macander et al., 2022; Nill et al., 2022), these studies did not directly assess accuracy of mapped changes. Here, we demonstrate that by inferring change from co-registered commercial satellite image pairs acquired several years apart (Derkacheva et al., 2025), we can successfully assess changes derived from time series of annual fractional land cover maps.

### 5.5 Future opportunities and applications

The mapping methodology developed in this study opens new opportunities to analyze the impacts of disturbance and climate change on Arctic land cover composition. Quantifying how landscape characteristics, climate drivers, vegetation composition, and disturbance severity shape vegetation change trajectories is essential for understanding the resilience of Arctic ecosystems and vegetation change feedbacks in the climate system (Foster et al., 2022). Comparing mapped change with long-term greening and browning patterns can also help disentangle signals driven by compositional shifts such as shrubification from other factors that lead to similar signals suggesting or reflecting increased productivity (Myers-Smith et al., 2020).

While our methods are designed to provide a foundation for future Arctic fractional land cover mapping, there is room for improvement. Our methodology would benefit from efforts to harmonize surface reflectance data across commercial satellite sensors, potentially reducing the amount of site-specific retraining required to produce high-quality land cover training and reference data and enabling efficient generation of training data over large areas. In parallel, advances in deep learning models, particularly those incorporating texture and multi-scale spatial context (Li et al., 2020; Wang et al., 2022), hold promise for improving the quality of training and reference data generated using high spatial resolution imagery. For the regression modeling step, we applied Random Forest regression, which is a widely used and relatively interpretable modeling approach. Emerging opportunities to enhance sub-pixel regression modeling include deep learning architectures such as neural network regression (Pham et al., 2024) and foundation models pre-trained on dense satellite time series including from HLS (Brown et al., 2025; Szwarcman et al., 2025), which produce embeddings estimated from multitemporal imagery that could complement the LSP-informed spectral-temporal features framework established here. Additionally, the development of a 10 m HLS product (Song et al., 2025) may improve mapping accuracy and detection of change. The relatively short temporal record from HLS in this study limits detection of long-term dynamics, but as additional years accumulate, algorithms that account for both gradual and abrupt transitions while mitigating noise will become increasingly useful for characterizing change (Baccini et al., 2017; Stanimirova et al., 2022).

Our mapping methodology differentiates low-stature vegetation, erect shrubs, and trees, vegetation classes linked to dominant land cover change processes in the Arctic including shrub expansion and disturbance-driven transitions. While our maps improve on previous mapping products, integration of information on vegetation structure from new missions such as NISAR may help to further constrain fractional estimates of erect shrubs (Beamish et al., 2020). Future mapping efforts should aim to better resolve PFTs including lichens, forbs, graminoids, dwarf shrubs, and erect shrubs to capture their different ecological roles, phenology, carbon cycling, and responses to climate warming (Beamish et al., 2020; Murphy et al., 2025). Hyperspectral remote sensing holds promise for distinguishing tundra PFTs (Yang et al., 2023, Yang et al., 2021), and future missions including SBG and Landsat NEXT hold promise for resolving these challenges at regional scales going forward.

While this study focuses on Arctic tundra, the core methodological framework of deriving spectral-temporal features from HLS LSP and training sub-pixel fractional cover models using commercial satellite imagery is not biome-limited. This study contributes to a growing body of research mapping sub-pixel land cover and land cover change using machine learning regression, an approach particularly suited to ecosystems where vegetation varies over short length scales (Hostert et al., 2003; Stanimirova et al., 2022). Spectral-temporal metrics have proven effective for fractional cover estimation across diverse ecosystems including savannas (Baumann et al., 2018), rangelands (Harkort et al., 2025), and mixed forests (Zhu et al., 2025), while the recent proliferation of commercial VHSR-Sat imagery provides a growing source of training and reference data, suggesting broad and increasing potential for adapting this workflow to fractional cover monitoring across spatially heterogeneous ecosystems.

## 6 Conclusion

We present a novel approach for mapping annual sub-pixel land cover fractions across Arctic tundra that integrates LSP-informed spectral-temporal features from dense HLS time series with high spatial resolution reference data from VHSR-Sat imagery. Our methodology enabled the first maps and assessments of annual fractional land cover and land cover change for Arctic tundra. This approach is readily scalable across the Arctic and can be extended in the future to study long-term Arctic land cover change. The resulting maps demonstrate improved performance relative to existing static and multitemporal map products, and quantify prediction uncertainty at the pixel level, improving interpretation and uncertainty propagation in downstream modeling applications. These maps can support a wide range of ecological inquiries, including investigations of shrub expansion; associated changes and feedbacks in productivity, carbon storage, and surface energy balance; post-disturbance recovery trajectories; and climate-vegetation feedbacks. By leveraging information from multiple sensors that measure phenomenon at different spatial and temporal scales, our approach establishes a practical framework for consistent land cover monitoring, an essential step toward disentangling the drivers, patterns, and consequences of land cover change during a critical time of climate change and disturbance intensification across the Arctic.

## CRediT authorship contribution statement

**Xiaoran Zhu:** Writing – review & editing, Writing – original draft, Visualization, Validation, Supervision, Software, Resources, Project administration, Methodology, Investigation, Funding acquisition, Formal analysis, Data curation, Conceptualization. **Jonathan A. Wang:** Writing – review & editing, Funding acquisition. **Oliver Sonnentag:** Writing – review & editing, Data curation. **Isla H. Myers-Smith:** Writing – review & editing. **Daryl Yang:** Writing – review & editing, Data curation. **Kathleen M. Orndahl:** Writing – review & editing, Data curation. **Leon Nill:** Writing – review & editing, Data curation. **Antoine Caron-Guay:** Writing – review & editing, Data curation. **Etienne Laliberté:** Writing – review & editing, Data curation. **Mark A. Friedl:** Writing – review & editing, Supervision, Funding acquisition, Conceptualization.

## Declaration of competing interest

The authors declare that they have no known competing financial interests or personal relationships that could have appeared to influence the work reported in this paper.

## Data Availability

Data will be made available on request.

## Appendix A Supplementary data

Supplementary material Supplementary methods, tables, and figures supporting the main text
