# Supplementary material for: Improved mapping of Arctic fractional land cover and land cover change from multi-resolution optical remote sensing
Source: Remote Sens Environ. 2026 Oct;344:115511. doi: 10.1016/j.rse.2026.115511 (PMC13426488; doi:10.1016/j.rse.2026.115511)
Supplement: Supplementary file 1 [file mmc1.docx]

**Supplemental Methods**

**S1. Sampling stable land cover pixels at change assessment sites for the comparison of spectral feature collection methods**

At each change assessment site, very high spatial resolution commercial satellite (VHSR-Sat) image pairs were co-registered to each other as well as to HLS imagery following the procedure described in Section S2 below (“VHSR-Sat imagery processing workflow”). Next, a random sample of 30-m pixels were selected at each site and manually reviewed for a total of 900 stable samples (300 from each site) where land cover showed no change between 2016 and 2024. Then, for each pixel-year we collected the reflectances of the six visible, near infrared, and shortwave infrared bands from HLS corresponding to the timing of mid-green-up, peak EVI2, and mid-green-down, comprising the HLS synthetic spectra derived from LSP. Similarly, we calculated fixed-window seasonal composites for each of the spectral bands from HLS and Landsat alone. The spring, summer, and fall windows were defined using day-of-year ranges 100 – 179, 180 – 220, and 221 – 300. To calculate composite spectral values, we used the median spectral values during the compositing period for each band. Composites over the spring, summer, and fall were compared against synthetic spectra from mid-green-up, peak EVI2, and mid-green-down respectively.

To quantify interannual variability of spectral values at the stable land cover pixels, we calculated the standard deviations of spectral reflectance values across time at each pixel. Lower standard deviation signifies greater year-to-year stability of the spectral features associated with that pixel’s fractional land cover mixture. Comparisons of spectral variability across time were conducted for each band by performing paired t-tests to test for significant differences in the average standard deviations for HLS synthetic versus HLS composite and Landsat composite spectra.

**S2. VHSR-Sat imagery processing workflow**

Each of the nine training and five assessment images was processed using the orthorectification toolbox from the Polar Geospatial Center (PGC, University of Minnesota, 2025) and the ASTER Global Digital Elevation Map (NASA/METI/AIST/Japan Spacesystems and U.S./Japan ASTER Science Team, 2019) and downloaded through the NASA Commercial Satellite Data Acquisition (CSDA) program.

We mitigated issues with mis-registration by co-registering each VHSR-Sat image to HLS imagery using phase cross-correlation implemented with the scikit-learn package (version 1.4.2) in Python 3.11.4. (Guizar-Sicairos et al., 2008), which identified the displacement between each upscaled VHSR-Sat image and the corresponding HLS scene acquired as close as possible to the date of peak EVI2 from the same year. The detected sub-pixel offsets were used to align the 2-m VHSR-Sat image to the HLS scene.

**S3. VHSR-Sat imagery classification and assessment**

Classification models for each training site map were estimated using a minimum of 500 randomly selected training pixels that were manually interpreted from 50 cm pansharpened imagery. The imagery was classified at 2-m resolution, and training pixels were assigned to a land cover class if it comprised greater than 50% of the 2-m pixel; if no cover class exceeded 50% by area, the training point was discarded. In addition to the multispectral VHSR-Sat imagery, elevation, slope, and aspect were derived from ArcticDEM from the Polar Geospatial Center (Porter et al., 2023) and provided additional features useful for differentiating between land cover types and topographic shadows. We used random forest classification with 200 trees and a minimum leaf sample size of 5 to predict the land cover type in each 2-m pixel. Maps were generated using an iterative procedure, where for each iteration, each map was assessed qualitatively and quantitatively, and training samples were added to underrepresented and frequently misclassified classes.

To assess the accuracy of the site maps, an independent sample of 500 (or more) pixels were selected and manually interpreted at each site. Reference pixels were selected using an area-based stratified random sample following community established ‘good practices’ (Olofsson et al., 2014; Tyukavina et al., 2025). To ensure representation of rare classes, each reference set for each site was required to include a minimum of ten pixels for each land cover class. Confusion matrices and user’s and producer’s accuracies were compiled for each land cover class (Tables S2 – S15).

User’s accuracies for the two primary vegetation classes of interest, low vegetation and shrubs, ranged from 89.3 – 99.2% and 50 – 95.6%, respectively, and producer’s accuracies ranged from 86 – 99% and 55.3 – 97.2%, respectively (Tables S2 – S15). Sites with low shrub accuracies (< 65%) were rare and are from three sites where shrubs comprised < 5% of the total mapped area, leading to accuracies sensitive to class imbalance.

**S4. Protocol for reconciling land cover classes in independent reference maps from UAV data**

The five UAV-derived reference datasets provided a robust basis for assessing fractional cover mapping accuracy. In four datasets, land cover classes could be directly cross-walked to the classification system used in our mapping framework (Table S18). However, in the Orndahl et al. (2022) dataset, which represents < 1% of assessment points, shrubs were categorized as deciduous or evergreen, without distinguishing by height between erect and dwarf shrubs. Because deciduous shrubs can have both dwarf and upright forms while evergreen shrubs are exclusively of dwarf form in the Arctic (Walker et al., 2005), we could not reliably disaggregate this class into our low vegetation and shrub classes. In our assessments, we consider their deciduous shrubs class as all erect shrubs (shrub cover in this study). The full set of cross-walking decisions is provided in Table S18. Each mapped pixel from each reference map was used in accuracy assessments of the final fractional cover maps.

We also note that the UAV-derived reference data are model-based products from supervised classification rather than direct field measurements. However, several factors support their validity as assessment data. The UAV imagery resolution (≤ 0.2 m) is ≥ 150 times finer than the 30-m maps. Aggregating UAV pixels to calculate fractional cover within each 30-m pixel reduces the impact of individual misclassifications through spatial averaging, although we note that spatially autocorrelated errors can still lead to biases in fractional aggregates. The map products achieved good overall accuracies in their original studies (75 – 92%). Additionally, using five independent UAV datasets across different regions minimizes systematic biases from any single product. Because these high-quality UAV-derived maps can be scaled to directly match the 30-m grid of our maps, they provide a practical approach for landscape-scale validation at the spatial extent and resolution required for rigorous statistical assessment.

**S5. Protocol for comparing maps and accuracies with existing fractional cover data products**

We compared the Nill et al. (2022) map to our results from 2017, a central year in their 2015 – 2020 product. We compared the Macander et al. (2022) map for circa-2020 to our 2020 results, and the He et al. (2019) map for circa-2015 to our results from 2016.

A major challenge in comparing datasets is differences in land cover class definitions across the land cover products, which made cross-walking plant functional types imperfect in some cases. For the Nill et al. (2022) map, there were no issues with cross-walking classes, and we only combined the herbaceous and lichen classes into a single class corresponding to our low vegetation class. For the Macander et al. (2022) map, we grouped the evergreen shrub, graminoids, forbs, and light macrolichen classes into low vegetation. We cross-walked the deciduous shrub class to our shrub class. Although there are dwarf deciduous species in the Arctic, we could not separate this out into our low vegetation class as the Macander et al. (2022) product does not separate these species from erect deciduous shrubs. For the He et al. (2019) map, we combined herbaceous and nonvascular classes into the low vegetation class, but their woody class included all woody shrubs including dwarf and erect forms (Table S19).

Due to the differences in land cover classes and imperfect cross-walking, we do not expect a one-to-one relationship between predicted fractional cover values for each cross-walked class, especially for the Macander et al. (2022) and He et al. (2019) products. However, in both cases, given their cross-walked shrub class includes what this study considers low vegetation, we would expect slight overprediction of shrub cover and underprediction of low vegetation cover in their product compared to what this study considers shrubs and low vegetation respectively across all cover fractions (0 – 100%). Deviations in the other direction in the comparisons can only be attributed to errors in either the existing product or this study. Similar conclusions can be drawn regarding assessments as the assessment data were cross-walked to the land cover classes defined in this study. Therefore, plots comparing cover from existing products to observed cover should not be treated as “true assessments” but rather comparisons that can provide qualitative information on mapping accuracies and errors.

We also note that the Macander et al. (2022) product was trained partly on UAV-based maps from Orndahl et al. (2022), making the comparison not fully independent. However, these data represent a small portion of both our assessment data and the training data used in Macander et al. (2022) (0.37% of our assessment data and < 2.91% of the Macander et al. (2022) training data). This minimal overlap does not appear to significantly affect the UAV-based assessment results.

**Supporting Tables**

**Table S1.** Reference sites where very high spatial resolution imagery and/or maps were used to train and assess our fractional land cover maps. Reference maps were scaled to 30-m fractional cover and used for the training and assessment of the fractional land cover maps. Site IDs (column ID) are included for training and assessment maps derived from very high spatial resolution satellite (VHSR-Sat) imagery and correspond to site numbers in the accuracy assessments (Tables S2 – S15). The bottom five assessment maps are from independent UAV data. Maps were not produced from the six change assessment images. These were instead used to interpret change within sampled pixels to assess mapped change. Latitude and longitude are provided for the centroid of each site.

| **Train/**  **Assess** | **ID** | **Image/map name** | **Source** | **Lat** | **Lon** | **Level 3 ecoregion(s)** | **Year** | **Spatial resolution** |
| --- | --- | --- | --- | --- | --- | --- | --- | --- |
| Training | 1 | WV02_20160803 | WorldView-2 | 67.9021 | -161.9875 | Arctic Foothills | 2016 | 2 m |
|  | 2 | GE01_20190723 | GeoEye-1 | 69.3518 | -150.3082 | Arctic Foothills | 2019 | 2 m |
|  | 3 | WV02_20200822 | WorldView-2 | 69.9489 | -145.9879 | Arctic Foothills | 2020 | 2 m |
|  | 4 | WV03_20230815 | WorldView-3 | 69.1338 | -157.8326 | Arctic Foothills | 2023 | 2 m |
|  | 5 | GE01_20180901 | GeoEye-1 | 66.0004 | -161.7379 | Seward Peninsula | 2018 | 2 m |
|  | 6 | WV02_20190820 | WorldView-2 | 64.8524 | -164.6162 | Seward Peninsula | 2019 | 2 m |
|  | 7 | WV03_20210804 | WorldView-3 | 64.6750 | -165.9647 | Seward Peninsula | 2021 | 2 m |
|  | 8 | WV03_20210712 | WorldView-3 | 68.5865 | -149.3158 | Brooks Range | 2021 | 2 m |
|  | 9 | GE01_20220803 | GeoEye-1 | 67.4667 | -157.5770 | Brooks Range | 2022 | 2 m |
| Assessment | 10 | GE01_20170802 | GeoEye-1 | 69.6361 | -139.2840 | Arctic Foothills | 2017 | 2 m |
|  | 11 | WV02_20180814 | WorldView-2 | 67.3275 | -163.1015 | Arctic Foothills | 2018 | 2 m |
|  | 12 | WV02_20200710 | WorldView-2 | 69.4539 | -154.7533 | Arctic Foothills | 2020 | 2 m |
|  | 13 | WV03_20240730 | WorldView-3 | 66.0578 | -163.5320 | Seward Peninsula | 2024 | 2 m |
|  | 14 | GE01_20210721 | GeoEye-1 | 68.7387 | -138.2369 | Brooks Range | 2021 | 2 m |
|  |  | Greaves_2019 | Greaves et al. (2019) | 68.6231 | -149.4672 | Brooks Range | 2013 | 20 cm |
|  |  | Fraser_2016 | Fraser et al. (2016) | 69.3658 | -133.0350 | Amundsen Plains | 2015 | 1 cm |
|  |  | Orndahl_2022* | Orndahl et al. (2022) | 68.6873 | -138.9745 | Arctic Foothills, Brooks Range | 2019 | 0.76 cm |
|  |  | Yang_2023 | Yang et al. (2023) | 65.0610 | -165.2000 | Seward Peninsula | 2019 | 1.5 cm |
|  |  | Caron-Guay_2025 | Caron-Guay et al. (2025) | 68.7435 | -133.5026 | Amundsen Plains | 2023 | 1.6 cm |
| Change assessment site 1 |  | WV02_20160713 | WorldView-2 | 64.5167 | -165.2638 | Seward Peninsula | 2016 | 2 m |
|  |  | WV03_20240722 | WorldView-3 |  |  | Seward Peninsula | 2024 | 2 m |
| Change assessment site 2 |  | WV02_20160714 | WorldView-2 | 69.7365 | -143.6590 | Arctic Foothills | 2016 | 2 m |
|  |  | WV02_20140805 | WorldView-2 |  |  | Arctic Foothills | 2024 | 2 m |
| Change assessment site 3 |  | WV03_20160824 | WorldView-3 | 65.9758 | -135.1625 | Brooks Range | 2016 | 2 m |
|  |  | WV03_20240807 | WorldView-3 |  |  | Brooks Range | 2024 | 2 m |

*Includes multiple site maps; pixels were aggregated by source as opposed to site for the assessment plots (Fig. 6)

**Table S2.** Area-based accuracy assessment for Site 1 land cover map, which was sampled for fractional cover model training. Site number and ecoregion name (underneath) on the top-left refer to the ID and Level 3 ecoregion(s) columns, respectively, in Table S1.

**Table S3.** Area-based accuracy assessment for Site 2 land cover map, which was sampled for fractional cover model training. Site number and ecoregion name (underneath) on the top-left refer to the ID and Level 3 ecoregion(s) columns, respectively, in Table S1.

**Table S4.** Area-based accuracy assessment for Site 3 land cover map, which was sampled for fractional cover model training. Site number and ecoregion name (underneath) on the top-left refer to the ID and Level 3 ecoregion(s) columns, respectively, in Table S1.

**Table S5.** Area-based accuracy assessment for Site 4 land cover map, which was sampled for fractional cover model training. Site number and ecoregion name (underneath) on the top-left refer to the ID and Level 3 ecoregion(s) columns, respectively, in Table S1.

**Table S6.** Area-based accuracy assessment for Site 5 land cover map, which was sampled for fractional cover model training. Site number and ecoregion name (underneath) on the top-left refer to the ID and Level 3 ecoregion(s) columns, respectively, in Table S1.

**Table S7.** Area-based accuracy assessment for Site 6 land cover map, which was sampled for fractional cover model training. Site number and ecoregion name (underneath) on the top-left refer to the ID and Level 3 ecoregion(s) columns, respectively, in Table S1.

**Table S8.** Area-based accuracy assessment for Site 7 land cover map, which was sampled for fractional cover model training. Site number and ecoregion name (underneath) on the top-left refer to the ID and Level 3 ecoregion(s) columns, respectively, in Table S1.

**Table S9.** Area-based accuracy assessment for Site 8 land cover map, which was sampled for fractional cover model training. Site number and ecoregion name (underneath) on the top-left refer to the ID and Level 3 ecoregion(s) columns, respectively, in Table S1.

**Table S10.** Area-based accuracy assessment for Site 9 land cover map, which was sampled for fractional cover model training. Site number and ecoregion name (underneath) on the top-left refer to the ID and Level 3 ecoregion(s) columns, respectively, in Table S1.

**Table S11.** Area-based accuracy assessment for Site 10 land cover map, which was sampled for fractional cover model assessment. Site number and ecoregion name (underneath) on the top-left refer to the ID and Level 3 ecoregion(s) columns, respectively, in Table S1.

**Table S12.** Area-based accuracy assessment for Site 11 land cover map, which was sampled for fractional cover model assessment. Site number and ecoregion name (underneath) on the top-left refer to the ID and Level 3 ecoregion(s) columns, respectively, in Table S1.

**Table S13.** Area-based accuracy assessment for Site 12 land cover map, which was sampled for fractional cover model assessment. Site number and ecoregion name (underneath) on the top-left refer to the ID and Level 3 ecoregion(s) columns, respectively, in Table S1.

**Table S14.** Area-based accuracy assessment for Site 13 land cover map, which was sampled for fractional cover model assessment. Site number and ecoregion name (underneath) on the top-left refer to the ID and Level 3 ecoregion(s) columns, respectively, in Table S1.

**Table S15.** Area-based accuracy assessment for Site 14 land cover map, which was sampled for fractional cover model assessment. Site number and ecoregion name (underneath) on the top-left refer to the ID and Level 3 ecoregion(s) columns, respectively, in Table S1.

**Table S16.** Comparison of the VHSR-Sat derived 30-m fractional cover map at Site 8 (Table S8) with an overlapping independent reference map (Greaves et al., 2019; Table S1). Errors and average biases are expressed relative to the independent reference data as observed/truth. The tree class is not included as there are no trees at this site. Details for cross-walking land cover classes from the Greaves et al. (2019) product are provided in Table S18.

| **Class** | **R^2^** | **MAE** | **RMSE** | **Bias** |
| --- | --- | --- | --- | --- |
| Water | 0.52 | 0.49% | 2.41% | 0.26% |
| Barren | 0.88 | 2.45% | 7.5% | 0.88% |
| Low vegetation | 0.74 | 7.55% | 14.18% | -0.89% |
| Shrub | 0.57 | 5.64% | 12.09% | -0.28% |

**Table S17.** All features used in model training. Harmonized Landsat and Sentinel-2 (HLS) bands, abbreviations, and bandwidths are as follow: Blue (B; 0.45 – 0.51 µm), Green (G; 0.53 – 0.59 µm), Red (R; 0.64 – 0.67 µm), Near infrared (NIR; 0.85 – 0.88 µm), Short-wave infrared 1 (SWIR1; 1.57– 1.65 µm), and Short-wave infrared 2 (SWIR2; 2.11 – 2.29 µm). The two-band Enhanced Vegetation Index (EVI2) is calculated as $2.5\cdot\frac{NIR - R}{NIR + R + 1}$. Tasseled cap greenness (TCG), Tasseled cap greenness (TCW), and Tasseled cap greenness (TCB) come from Baig et al. (2014).

| **Category** | **Feature** |
| --- | --- |
| Mid-green-up spectra | B |
|  | G |
|  | R |
|  | NIR |
|  | SWIR1 |
|  | SWIR2 |
| Mid-green-up spectral indices | EVI2 |
|  | TCG |
|  | TCW |
|  | TCB |
| Peak spectra | B |
|  | G |
|  | R |
|  | NIR |
|  | SWIR1 |
|  | SWIR2 |
| Peak spectral indices | EVI2 |
|  | TCG |
|  | TCW |
|  | TCB |
| Mid-green-down spectra | B |
|  | G |
|  | R |
|  | NIR |
|  | SWIR1 |
|  | SWIR2 |
| Mid-green-down spectral indices | EVI2 |
|  | TCG |
|  | TCW |
|  | TCB |
| Topographic indices | Elevation |
|  | Slope |
|  | Aspect |

**Table S18.** Independent reference sites with their original land cover classes along with cross-walked classes used in this study. These included vegetation communities as well as the final land cover classes used in fractional cover mapping. Most maps were assessed against the same year of reference data, but for the Greaves and Fraser maps, the assessed year is 2016, the closest mapping year to the data collection years.

| **Site** | **Assessed Year** | | **Data Year** | **Cross-walked class** | **Original class** |
| --- | --- | --- | --- | --- | --- |
| Greaves et al. (2019) | 2016 | | 2013 | Water | Water |
|  |  |  |  | Barren | Barren/Gravel pads |
|  |  |  |  |  | Rock/Infrastructure |
|  |  |  |  | Low vegetation | Dry exposed tundra |
|  |  |  |  |  | Dry sheltered tundra |
|  |  |  |  |  | Tussock tundra |
|  |  |  |  |  | Shrubby tussock tundra |
|  |  |  |  |  | Moist snowbed |
|  |  |  |  |  | Moist non-tussock tundra |
|  |  |  |  |  | Shrubby moist non-tussock tundra |
|  |  |  |  |  | Raised areas in wet tundra |
|  |  |  |  |  | Wet tundra |
|  |  |  |  |  | Low dense shrub (< 30 cm) |
|  |  |  |  | Shrub | Low to tall moist shrub |
|  |  |  |  |  | Tall shrub |
| Fraser et al. (2016) | 2016 | | 2015 | Water | Water |
|  |  |  |  | Barren | Soil and dark cryptogams |
|  |  |  |  | Low vegetation | Reindeer lichen |
|  |  |  |  |  | Mixed dwarf shrub heath |
|  |  |  |  |  | Moss |
|  |  |  |  |  | Wet graminoid |
|  |  |  |  |  | Sedge tussock |
|  |  |  |  | Shrub | Willow |
|  |  |  |  |  | Alder |
|  |  |  |  |  | Birch |
| Orndahl et al. (2022) | 2019 | | 2019 | Barren | Non-vegetated |
|  |  |  |  | Low vegetation | Bryophytes |
|  |  |  |  |  | Evergreen shrub |
|  |  |  |  |  | Forbs |
|  |  |  |  |  | Graminoids |
|  |  |  |  |  | Lichens |
|  |  |  |  | Shrub | Deciduous shrub |
|  |  |  |  | Tree | Trees |
| Yang et al. (2023) | 2019 | | 2019 | Barren | Non-vegetated surface |
|  |  |  |  | Low vegetation | Deciduous dwarf shrub |
|  |  |  |  |  | Evergreen shrub |
|  |  |  |  |  | Forb |
|  |  |  |  |  | Lichen |
|  |  |  |  |  | Dry graminoid |
|  |  |  |  |  | Moss |
|  |  |  |  |  | Wet graminoid |
|  |  |  |  | Shrub | Deciduous tall shrub alder |
|  |  |  |  |  | Deciduous tall shrub willow |
|  |  |  |  |  | Deciduous low shrub |
|  |  |  |  | Tree | Evergreen tree |
|  |  |  |  |  | Deciduous tree |
| Caron-Guay et al. (2025) | 2023 | 2023 | | Low vegetation | Lichen-dwarf shrub tundra |
|  |  |  |  |  | Tussock |
|  |  |  |  |  | Moss |
|  |  |  |  |  | Sedge |
|  |  |  |  | Shrub | Alder |
|  |  |  |  |  | Birch |
|  |  |  |  |  | Willow |

**Table S19.** Existing map products used in comparisons and comparative assessments with their original land cover classes along with cross-walked classes used in this study. Most maps were compared for the same period, but for the He et al. (2019) product, which was made for circa-2015, we compared with our results for 2016.

| **Map product** | **Period in map product** | **Year from this study** | **Cross-walked class** | **Original class in map product** |
| --- | --- | --- | --- | --- |
| Nill et al. (2022) | 2015 – 2020 | 2017 | Water | Water |
|  |  |  | Barren | Barren |
|  |  |  | Low vegetation | Lichen |
|  |  |  |  | Herbaceous |
|  |  |  | Shrub | Shrub |
|  |  |  | Tree | Coniferous |
| Macander et al. (2022) | 2020 | 2020 | Low vegetation | Light macrolichens |
|  |  |  |  | Forbs |
|  |  |  |  | Graminoids |
|  |  |  |  | Evergreen shrubs |
|  |  |  | Shrub | Deciduous shrubs |
|  |  |  | Tree | Broadleaf trees |
|  |  |  |  | Conifer trees |
| He et al. (2019) | Circa-2015 | 2016 | Low vegetation | Nonvascular |
|  |  |  |  | Herbaceous |
|  |  |  | Shrub | Woody |

**Table S20.** Results from two-way mixed-effects Intraclass Correlation Coefficients (ICC) across maps of standard deviation uncertainties from 2016 – 2023 for each land cover type indicate stable uncertainties across years.

| **Land cover type** | **ICC** |
| --- | --- |
| Water | 0.836 |
| Barren | 0.800 |
| Low vegetation | 0.734 |
| Shrub | 0.763 |
| Tree | 0.837 |

**Table S21.** Details of including how the reference data were collected and the modeling accuracies of each (left) and comparisons of assessments against all UAV-derived independent maps and all VHSR-Sat assessment maps (right). Overall accuracies for UAV-derived data are shown for the original classes, not cross-walked classes, as the assessment data were not available in all cases to refactor assessments.

| **Assessment dataset information** | | | |  | **Assessment of this study** | | | | |
| --- | --- | --- | --- | --- | --- | --- | --- | --- | --- |
| **Data type** | **Dataset** | **Model** | **Overall accuracy** |  | **Class** | **R^2^** | **MAE** | **RMSE** | **Bias** |
| UAV-derived reference data | Greaves et al., 2019 | Random Forest | 86% |  | Water | 0.77 | 0.7% | 4.7% | -0.5% |
|  | Fraser et al. (2016) | eCognition segementation, See5 decision trees | 82% |  | Barren | 0.51 | 7.1% | 14.1% | -0.8% |
|  | Orndahl et al. (2022) | Random Forest | 75% |  | L-Veg | 0.44 | 16% | 22.5% | 0.1% |
|  | Yang et al. (2023) | Random Forest | 86 – 92% |  | Shrub | 0.42 | 11.9% | 18.6% | 0.8% |
|  | Caron-Guay et al. (2025) | ResNet-101 | 90%* |  | Tree | 0.00 | 0.4% | 2.9% | 0.4% |
| VHSR-Sat assessment data | Site 10 | Random Forest | 95% |  | Water | 0.93 | 1.8% | 8.8% | 0.9% |
|  | Site 11 | Random Forest | 92% |  | Barren | 0.50 | 7.4% | 16.4% | 5.6% |
|  | Site 12 | Random Forest | 94% |  | L-Veg | 0.69 | 17.4% | 24.3% | -10% |
|  | Site 13 | Random Forest | 97% |  | Shrub | 0.47 | 12.2% | 19.4% | 3.4% |
|  | Site 14 | Random Forest | 92% |  | Tree | 0.39 | 0.7% | 3.6% | 0.0% |

*Manuscript in preparation.

**Supporting Figures**

**Fig. S1.** The density of clear HLS observations unobstructed by ice, snow, and cloud cover at each pixel in the study area for years 2016 – 2023.

**Fig. S2.** Harmonized Landsat and Sentinel-2 (HLS) synthetic spectra derived from land surface phenology (HLS synthetic) show significantly lower interannual variability for pixels with stable land cover than HLS or Landsat (L30) composites for most spectral-temporal features. Data in the figure are from 300 randomly selected HLS pixels with no land cover change identified at each of the three change assessment sites for a total of 900 stable pixels between 2016 and 2024. Significance levels (NS: *p* ≥ 0.05, *: *p* < 0.05, **: *p* < 0.01, ***: *p* < 0.001) from paired t-tests are displayed above each boxplot pair.

**Fig. S3.** Bias correction results for one set of 100 modeling runs, showing predicted versus observed values for each land cover type. Uncorrected comparisons are shown in the top plots, and bias-corrected comparisons are shown in the bottom plots.

**Fig. S4.** Fractional cover for 2020 for each mapped land cover type. L-veg stands for Low vegetation.

**Figure S5.** Assessment of predicted fractional cover maps against observed fractional cover from five very high spatial resolution satellite (VHSR-Sat) assessment sites (Fig. 1, Table S1) for (a) water, (b) barren, (c) tree, (d) low vegetation, and (e) shrub cover. A stratified random sample of 2,000 pixels was selected at each site. Each circle represents a site-bin where observed cover at each VHSR-Sat assessment map has been binned into 10% fractional cover bins from 0 – 100%. The position of the circle is determined by the means of observed and predicted cover within each site-bin, and the size corresponds to the number of data points (pixels). Regression statistics are calculated for all sampled data points.

**Figure S6.** Plots comparing fractional cover within each cross-walked land cover class between the Nill et al. (2022) product for the year 2017 and this study for the year 2017 (top); and assessments for both sets of results against unmanned aerial vehicle (UAV) -based maps at independent reference sites. The map shows the comparison study area where results overlapped outlined in magenta. The bar graph shows the number of pixels from each independent assessment site.

**Figure S7.** Plots comparing fractional cover within each cross-walked land cover class between the Macander et al. (2022) product for the year 2020 and this study for the year 2020 (top); and assessments for both sets of results against unmanned aerial vehicle (UAV) -based maps at independent reference sites. The map shows the comparison study area where results overlapped outlined in magenta. The bar graph shows the number of pixels from each independent assessment site.

**Figure S8.** Plots comparing fractional cover within each cross-walked land cover class between the He et al. (2019) product for the year 2015 and this study for the year 2016 (top); and assessments for both sets of results against unmanned aerial vehicle (UAV) -based maps at independent reference sites. The map shows the comparison study area where results overlapped outlined in magenta. The bar graph shows the number of pixels from each independent assessment site.

**Figure S9.** Correlation between the differenced Normalized Burn Ratio (dNBR) and percent change in barren cover for the 2019 Kugruk River fire #1 (a), 2019 Kaluktavik River fire (b), and 2017 Dietrich River fire (c) show a strong, positive relationship between magnitude of barren increase and burn severity. Differencing took place between the year before and the year after fire. Normalized burn ratio (NBR) was calculated from the peak synthetic spectra.

**Figure S10.** Comparisons of time series of total vegetation cover within fire perimeters at the same fire sites as Fig. 10 illustrate that our annual maps can better quantify and attribute pulse changes related to fire compared to the Macander et al. (2022) product for 2015 and 2020 (dash-dotted lines), which maps vegetation cover every five years. Shaded envelopes in the annual time series from this study correspond to average standard deviations from mapped uncertainties over the disturbed area, and shaded envelopes around the Macander et al. (2022) time series are average RSMEs provided with the product. The dashed red line indicates the fire year. We do not compare to the Nill et al. (2022) maps as there are no spatial overlaps at these sites.

**Figure S11.** Comparisons of time series of total vegetation cover within retrogressive thaw slumps (RTS) perimeters at the first two RTS sites from Fig. 11 illustrate that our annual maps can better quantify and attribute pulse changes related to RTS events compared to the Nill et al. (2022) maps for 2009 – 2014 and 2015 – 2020 (top) and Macander et al. (2022) maps for 2015 and 2020 (bottom; dash-dotted lines). Shaded envelopes in the annual time series from this study correspond to average standard deviations from mapped uncertainties over the disturbed area, and shaded envelopes around the Macander et al. (2022) time series are average RSMEs provided with the product. The Nill et al. (2022) maps do not provide uncertainty metrics. We do not compare at sites c and d from Fig. 11 as there are no spatial overlaps with the Nill et al. (2022) maps at these sites.

**Figure S12.** Repeat true color imagery from 2016 and 2024 at the three change assessment sites, overlaid with the predicted per-pixel fractional change rate for barren (top), water (middle), and shrub (bottom) cover as estimated using the Theil-Sen slope in annual fractional cover changes. All changes shown were significant (p < 0.05) based on the Mann Kendall test.

**References**

Guizar-Sicairos, M., Thurman, S.T., Fienup, J.R., 2008. Efficient subpixel image registration algorithms. Opt. Lett. 33, 156–158. doi:10.1364/OL.33.000156.

NASA/METI/AIST/Japan Spacesystems and U.S./Japan ASTER Science Team, 2019. ASTER Global Digital Elevation Model V003. NASA Land Processes Distributed Active Archive Center. doi:10.5067/ASTER/ASTGTM.003.

Polar Geospatial Center (PGC), University of Minnesota, 2025. PGC Imagery Utilities for Satellite Image Processing, Including Orthorectification. GitHub Repos. https://github.com/PolarGeospatialCenter/imagery_utils.
